# Predicting the Conformation of Organic Catalysts Grafted on Silica Surfaces with Different Numbers of Tethering Chains: The Silicopodality Concept

**DOI:** 10.1021/acs.jpcc.1c06150

**Published:** 2021-09-17

**Authors:** Ivana Miletto, Chiara Ivaldi, Enrica Gianotti, Geo Paul, Fabio Travagin, Giovanni Battista Giovenzana, Alberto Fraccarollo, Davide Marchi, Leonardo Marchese, Maurizio Cossi

**Affiliations:** † Dipartimento di Scienze e Innovazione Tecnologica (DISIT), Università del Piemonte Orientale, via T. Michel 11, I-15121 Alessandria, Italy; ‡ Dipartimento di Scienze del Farmaco (DSF), Università del Piemonte Orientale, L.go Donegani 2, I-28100 Novara, Italy; § CAGE Chemicals srl, Via Bovio 6, I-28100 Novara, Italy

## Abstract

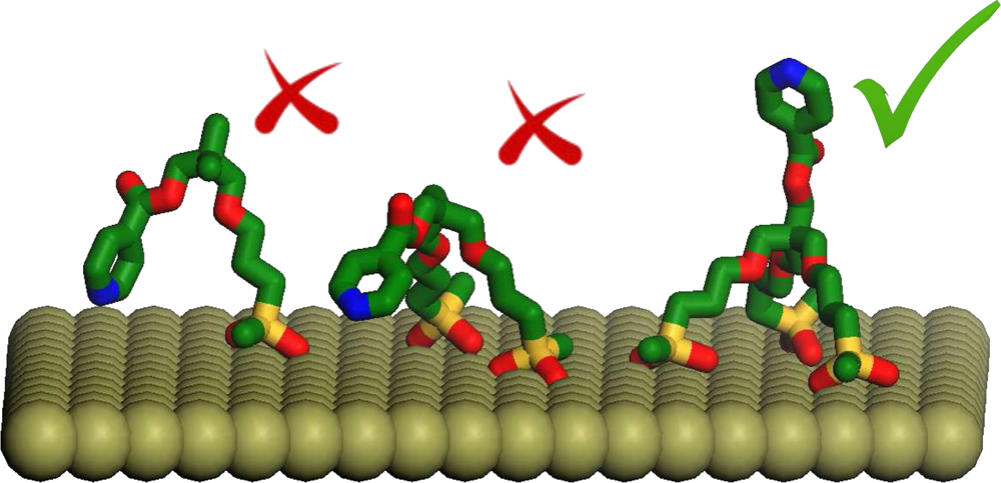

Hybrid catalysts are attracting much attention, since they combine the versatility and efficiency of homogeneous organic catalysis with the robustness and thermal stability of solid materials, for example, mesoporous silica; in addition, they can be used in cascade reactions, for exploring both organic and inorganic catalysis at the same time. Despite the importance of the organic/inorganic interface in these materials, the effect of the grafting architecture on the final conformation of the organic layer (and hence its reactivity) is still largely unexplored. Here, we investigate a series of organosiloxanes comprising a pyridine ring (the catalyst model) and different numbers of alkylsiloxane chains used to anchor it to the MCM-41 surface. The hybrid interfaces are characterized with X-ray powder diffraction, thermogravimetric analyses, Fourier-transform infrared spectroscopy, nuclear magnetic resonance techniques and are modeled theoretically through molecular dynamics (MD) simulations, to determine the relationship between the number of chains and the average position of the pyridine group; MD simulations also provide some insights about temperature and solvent effects.

## Introduction

Hybrid catalysts, formed by organic moieties anchored onto inorganic matrices, are interesting for their capacity to combine enormous varieties of organic functional groups with a solid support with superior mechanical and thermal stabilities.^1−11^ Such materials offer the advantages of homogeneous catalysis, as high yields, selectivities and possible stereoselectivity, along with those of heterogeneous catalysis, with easily separable products, reduced volume of solvents and so on.^12−17^ Even more interesting is the possibility of using active inorganic surfaces, for instance silica with suitable concentrations of silanol groups, or silica–alumina surfaces with Brönsted and Lewis acid sites, which can in turn act as cocatalysts in cascade reactions.^18−22^

Despite the great potential of hybrid heterogeneous catalysts, very little is known so far about the preferred conformations of the organic moieties grafted to the surface, and their influence on the catalytic activity. In particular, the active organic groups are likely more available for interactions with the substrates when they spend most of the time as far as possible from the surface, increasing the efficiency, while the tendency to lie down on the surface is expected to have the opposite effect.

Here, we consider an organic catalyst (a pyridine moiety) grafted to the silica surface through alkylsiloxane chains terminated by −Si(OR)_*n*_R_3–*n*_^′^, with *n* = 1–3, R, R′ = methyl and ethyl.^23−25^ The terminal −OR groups can condensate with surface silanols to form siloxane bridges binding the organosiloxane to amorphous or mesoporous silica or silica–alumina: the number of bridges formed by each chain depends on the number of alkoxy groups (*n* above) and on the surface silanol concentration.

**Figure 1:**
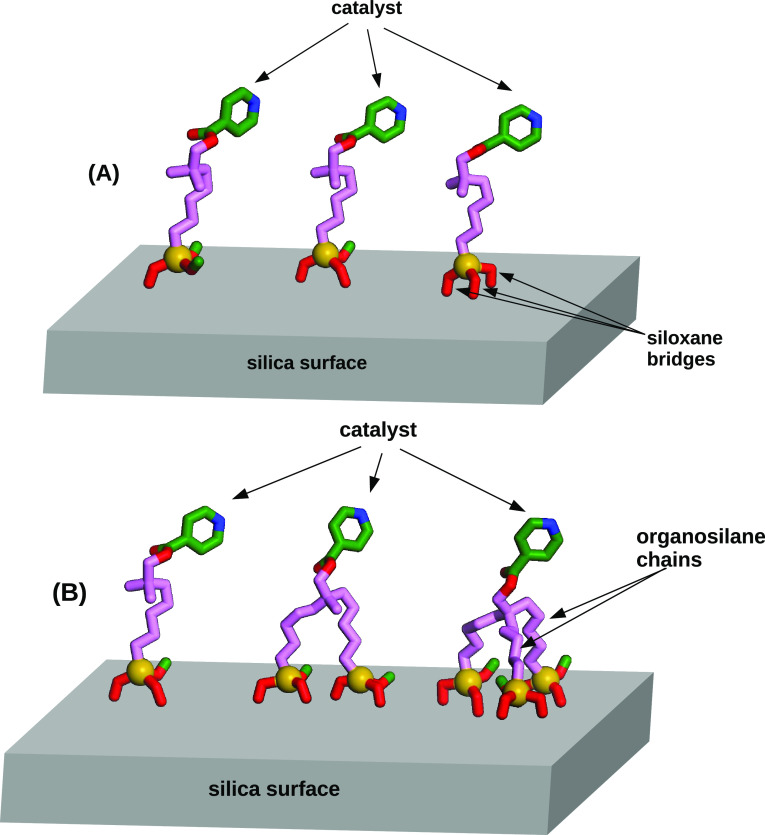
Scheme of the possible tethering architectures: (A), changing the number of siloxane bridges (dactyly); (B), changing the number of siloxane chains (podality).

We investigate how the design of the tethering unit affects the interface conformation, adopting a combined experimental/computational approach, which is particularly suited to deal with hybrid materials.^26^ Indeed, there are two structural parameters which can be easily varied during the organosiloxane synthesis, illustrated in Figure 1. The first parameter is the number of siloxane bridges formed by each chain: in ref (27), we proposed to call this parameter *silicodactyly* (from dactyls, the Greek word for fingers) and studied its effect on the conformation of the hybrid structures with a combination of experimental and computational techniques. The conclusion was that silicodactyly has a very little effect on the conformation of grafted organic chains, which tend to lie close to the silica most of the time, irrespective of the number of siloxane “fingers” used to grab the surface.

The second structural element that can be adjusted is the number of siloxane chains linked to the same organic catalyst, holding the active group on the silica surface. This number will be referred to as *silicopodality*, and in the following we study how it affects the conformation of the organic/silica interface, using a combination of theoretical modeling and experimental characterization.

**Figure 2:**
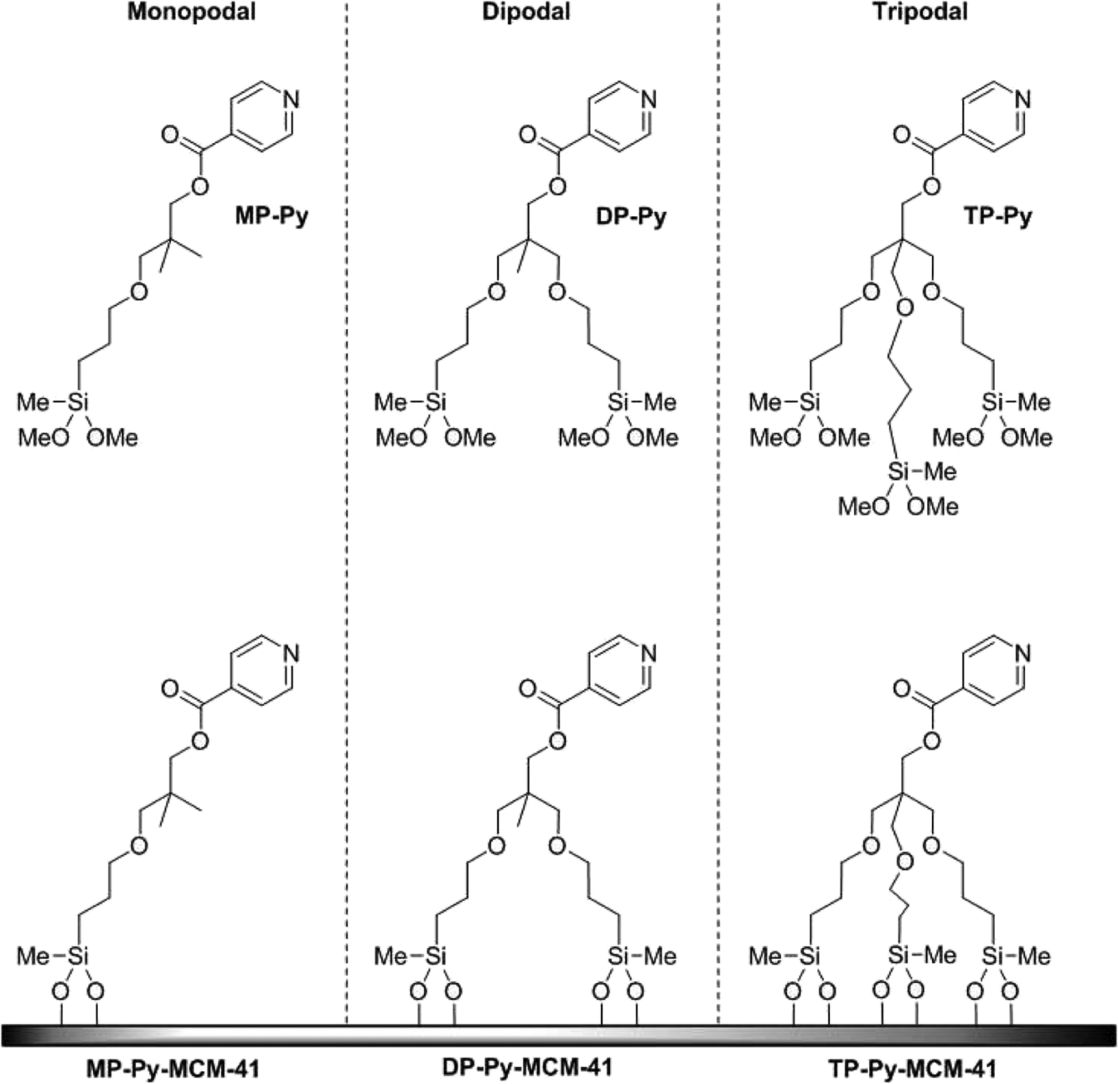
Chemical structure and acronym of the multipodal 4-pyridine derivatives isolated and grafted to the mesoporous silica.

This study concerns a series of pyridine-substituted derivatives, with different numbers of alkylsiloxane chains, illustrated in Figure 2. Here, the pyridine moiety represents the organic catalyst ( e.g., in acylation reactions), and it can also interact with silanols, revealing how close to the surface the active center lies.

Notably, all the tethering chains in the considered systems bear two −OMe residues, along with an unreactive methyl group, so that each chain can form two siloxane bridges with the surface (a structure called didactyl in ref (27)). We did not examine other tethering schemes, because the risk of monodactyl grafting is too low, while tri-dactyl structures are statistically unlikely, unless a very hydrophilic silica is used: anyway, as mentioned above, the effect of silicodactyly on the conformation of the organic/silica interface is very limited.^27^

Three pyridine derivatives, namely MP-Py, DP-Py, and TP-Py, bearing one, two, or three alkylsiloxane chains, respectively, were synthesized; MP-Py and TP-Py were grafted to MCM-41 ordered mesoporous silica, and characterized by Fourier-transform infrared (FT-IR) spectroscopy, X-ray powder diffraction (XRPD), thermogravimetric analysis (TGA), and solid-state nuclear magnetic resonance (SS-NMR) analyses. In addition, all the hybrid systems (MP-Py-MCM41, DP-Py-MCM41, and TP-Py-MCM41) were modeled theoretically with molecular dynamics (MD) simulations, comparing the results of the simulations with the experimental results.

## Materials and Methods

### Synthesis of the Multipodal Pyridine Derivatives

**Figure 3:**
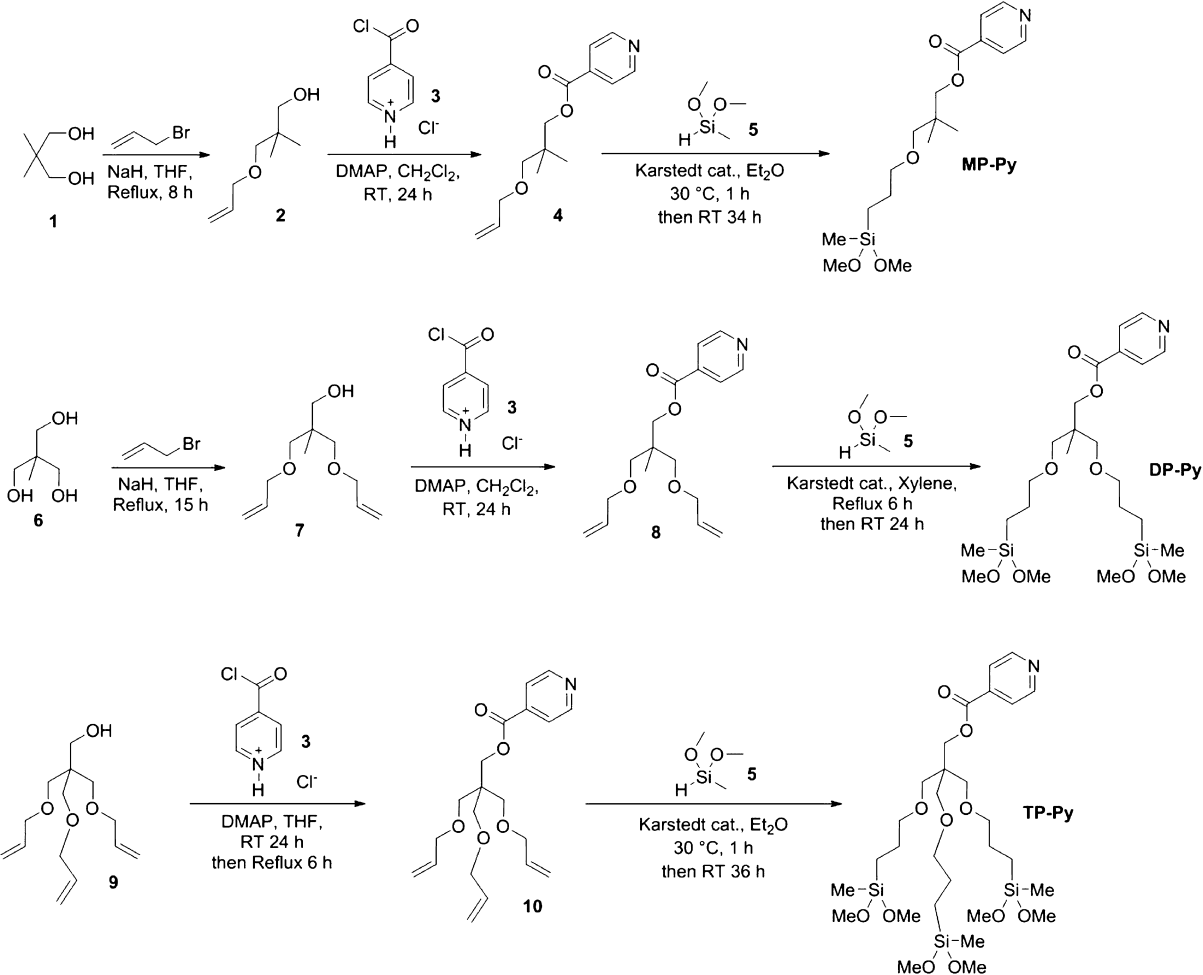
Synthesis of the multipodal pyridine-substituted derivatives.

The pyridine derivatives, bearing one to three alkylsiloxane chains for grafting to the silica surface, were prepared following the scheme shown in Figure 3.

The commercially available polyols **1** and **6** were used as the starting materials for the monopodal and dipodal derivatives. Mono and diallylation of these two polyols were accomplished through a classic Williamson synthesis, by treating the polyol with sodium hydride in tetrahydrofuran (THF), followed by reaction with allyl bromide. Separation of the mono (**2**) or diallyl derivative (**7**) from various allylated derivatives was achieved by column chromatography.

Compound **7** is the starting material for the preparation of the tripodal derivative. It is commercially available as a mixture of allylated pentarhythritols, enriched in the triallyl derivative (assay 70%), from which the pure pentaerythritol triallyl ether (**7**) may be obtained by simple displacement chromatography.^28^ The pyridine ring was introduced in the structure through an esterification step, taking advantage of the purposefully residual alcoholic group in each of the allylated derivatives (**2**, **7**, and **9**).

Isonicotinic acid was activated by conversion into the corresponding acid chloride **3**, and isolated as the hygroscopic crystalline hydrochloride. Reaction of **3** with the allylated derivatives **2**, **7**, and **9** provided the corresponding esters **4**, **8**, and **10**, respectively. Then, the mono-, di-, and tripodal esters were hydrosilylated with methyldimethoxysilane (**5**). The latter reacts with alkenes in the presence of a platinum catalyst (Karstedt catalyst),^29^ adding to the double bond with anti-Markovnikov selectivity, and leading to the introduction of the didactyl reactive silicon-based functional group and the desired monopodal, dipodal, and tripodal didactyl derivatives.

Solvents, MCM-41, and starting materials were purchased from Merck or TCI and used without further purification. Pentaerythritol triallyl ether (**9**) was purchased from Sigma-Aldrich at a 70% technical grade and it was purified by chromatography through a silica column (Pet/EtOAc 5:1). Sodium hydride (60% in mineral oil) was suspended and stirred in petroleum ether and then the supernatant was poured. This procedure was repeated three times to remove the mineral oil. Isonicotinoyl chloride hydrochloride (**3**) was prepared according to the literature procedure.^30^

^1^H and ^13^C NMR spectra were recorded at 300 MHz on a JEOL Eclipse ECP300 spectrometer or at 400 MHz on a Bruker AVANCE Neo 400 instrument. Chemical shifts are reported in ppm with the protic impurities of the deuterated solvent as the internal reference. Mass spectra were obtained with a Thermo Finnigan LCQ-Deca XP-PLUS ion trap spectrometer equipped with an electrospray source. TLC was performed with silica gel (MN Kieselgel 60F254) and visualized by UV or sprayed with Dragendorff reagent or alkaline KMnO_4_. Column chromatography was carried out on Macherey-Nagel silica gel 60 (0.063–0.200 mm).

The details of the synthesis reactions and of the spectroscopic characterization of products are reported in the Supporting Information.

### Synthesis of the Hybrid Materials

MCM-41 (0.3 g), dried overnight at 100 °C, was suspended in dry toluene (30 mL) and heated at 120 °C under stirring. Pyridine derivatives (0.0579 mmol) were added dropwise and the mixture was refluxed for 18 h; the reaction solution was filtered and washed with toluene. The white solid obtained was dried at 80 °C overnight.

### Physicochemical Characterization

X-ray powder diffraction (XRPD) patterns were recorded using an ARL XTRA48 diffractometer with Cu Kα radiation (*l* = 1.54062 Å). Diffractograms were recorded at room temperature in the high-angle (2θ = 5–5°) and low-angle (2θ = 1–10°) range with a rate of 1.0° min^–1^. The X-ray profiles at low angles were collected with narrower slits.

Thermogravimetric analyses (TGA) were carried out on a SETSYS Evolution TGA–DTA/DSC thermobalance, under argon flow at a gas flow rate of 100 mL min^–1^. The samples were heated from 30 to 1000 °C at a heating rate of 5 °C min^–1^.

Before recording the FT-IR and SS-NMR spectra, all the samples were outgassed at 150 °C for 1 h to remove physisorbed water. FT-IR analyses of the self-supporting pellets were performed under vacuum conditions (residual pressure <10^–4^ mbar) using a Bruker Equinox 55 spectrometer equipped with a pyroelectric detector (DTGS type) with a resolution of 4 cm^–1^. FT-IR spectra were normalized with respect to the pellet weight. Variable temperature FT-IR measurements were performed in the 30–500 °C temperature range, using a specifically designed cell permanently connected to the vacuum line.

Solid-state NMR spectra were acquired on a Bruker AVANCE III 500 spectrometer and using a wide bore 11.7 T magnet with operational frequencies 500.13, 99.35, and 125.77 MHz for ^1^H, ^29^Si, and ^13^C, respectively. A 4 mm triple resonance probe with magic angle spinning (MAS) was employed in all the experiments and the samples were packed on a Zirconia rotor and spun at a MAS rate between 10 and 15 kHz. The magnitude of radio frequency (RF) fields was 100 and 42 kHz for ^1^H and ^29^Si, respectively. For the ^13^C and ^29^Si cross polarization (CP) MAS experiments, the RF fields of 55 and 28 kHz were used for initial proton excitation and decoupling, respectively. During the CP period, the ^1^H RF field was ramped using 100 increments, whereas the ^13^C/^29^Si RF fields were maintained at a constant level. During the acquisition, the protons are decoupled from the carbons/silicons by using a two-pulse phase-modulated decoupling scheme. The relaxation delay, *d*_1_, between accumulations was 5 s for ^1^H MAS and ^13^C/^29^Si CPMAS NMR. All chemical shifts were reported by using the δ scale and are externally referred to TMS. ^1^H MAS NMR spectra were deconvoluted for quantitative interpretation of overlapping peaks.^31^

### Computational Modeling

MD simulations were performed in the canonical (*n*, *V*, *T*) ensemble at 298 and 353 K (using a Langevin thermostat to maintain a constant temperature),^32^ either in vacuum or in solution of dimethylformamide (DMF) or THF; for the simulations in liquid phase, a box of solvent was previously prepared and equilibrated to the experimental density, then a suitable number of solvent molecules were deleted to accommodate the hybrid systems. In all the MD calculations, an equilibration step of 0.5 ns was performed, followed by a 1 ns production run, using the LAMMPS simulation package.^33^ The Conjugate Gradients algorithm was used, with an energy tolerance of 10^–3^ kcal/mol, and a force tolerance of 0.5 kcal/(mol Å). Atom–atom parameters were taken from the universal force field (UFF),^34^ and atomic partial charges were generated by the QEq equilibration method, with a 10^–6^ e convergence. Coulombic interactions were computed with standard Ewald summation with a 10^–6^ kcal/mol accuracy, and van der Waals interactions were calculated with 6-12 Lennard-Jones function, using a 20 Å cutoff and parameters extracted from UFF.

Three models of pyridine-substituted derivatives were defined, as specified in the Introduction, and grafted on a silica slab with a thickness of 13.96 Å and a silanol surface density of 2.4 nm^–2^. Periodic boundary conditions were applied, with a 23.34 Å × 26.55 Å × 50.00 Å simulation box, large enough to exclude image interactions; a picture of the simulation box and the coordinates of the silica slab are provided in the Supporting Information.

## Results and Discussion

### XRPD and TGA

To confirm that the grafting procedure does not alter the inorganic support structure, X-ray powder diffraction was performed on hybrid materials as well as on MCM-41. Both MP-Py-MCM-41 and TP-Py-MCM-41 exhibit all the characteristic reflections of hexagonally ordered MCM-41,^35^ though less intense for the anchoring procedure lowering the structural order (patterns are shown in the Supporting Information).

TGA and differential thermogravimetry (DTG) provided insights on the thermal stability and hydrophilicity of the hybrid materials: as reported in Table 1 and illustrated in the Supporting Information, over the whole temperature range, the weight loss is slightly lower for TP-Py-MCM-41, indicating that a smaller amount of pyridine derivatives is bound to the surface, possibly because the tripodal arrangement is statistically less favorite.

**Table 1:** Weight Losses (%) in plain MCM-41, Monopodal and Tripodal Hybrids

*T* (°C)	MCM-41	MP-Py-MCM-41	TP-Py-MCM-41
30–100	3.1	3.1	3.5
350–550		6.4	5.5

Both hybrids and plain MCM-41 undergo a first weight loss in the range 30–100 °C, due to physisorbed water evaporation: the total loss for *T* < 100 °C is slightly higher for TP-Py-MCM-41, revealing a higher hydrophilicity for this material, in agreement with the previous observation of a smaller organic coverage. A second weight loss is detected around 350–550 °C for both the hybrids, attributed to the decomposition of grafted pyridine derivatives: in this case, MP-Py-MCM-41 loses more organics than its tripodal counterpart (around 6.4 and 5.5%, respectively).

Moreover, in the latter temperature range, DTG shows two contributions for MP-Py-MCM-41, at 380 and 465 °C, while for TP-Py-MCM-41 a single loss at 465 °C is present. This can be interpreted by observing that the weight ratio of pyridine rings over organosiloxane chains is larger in the monopodal than in the tripodal hybrids: in MP-Py-MCM-41, the first loss at 380 °C can be attributed to the decomposition of the pyridine group, and the second to the loss of the remaining organic chains, while in TP-Py-MCM-41, the pyridine contribution is less appreciated and a single loss is detected for the whole organic layer.

### FT-IR

**Figure 4:**
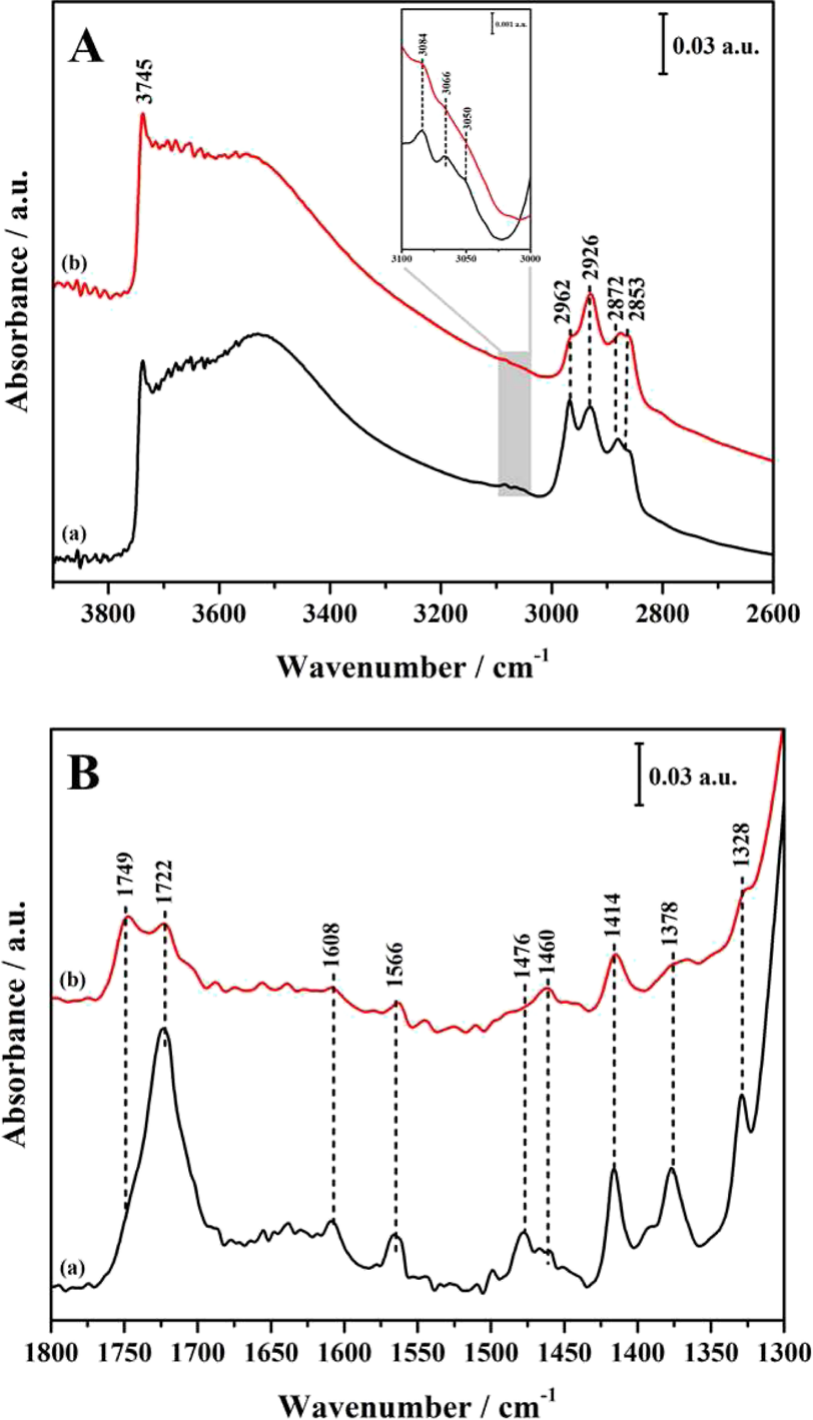
FT-IR spectra of MP-Py-MCM-41 (a, black) and TP-Py-MCM-41 (b, red).

**Figure 5:**
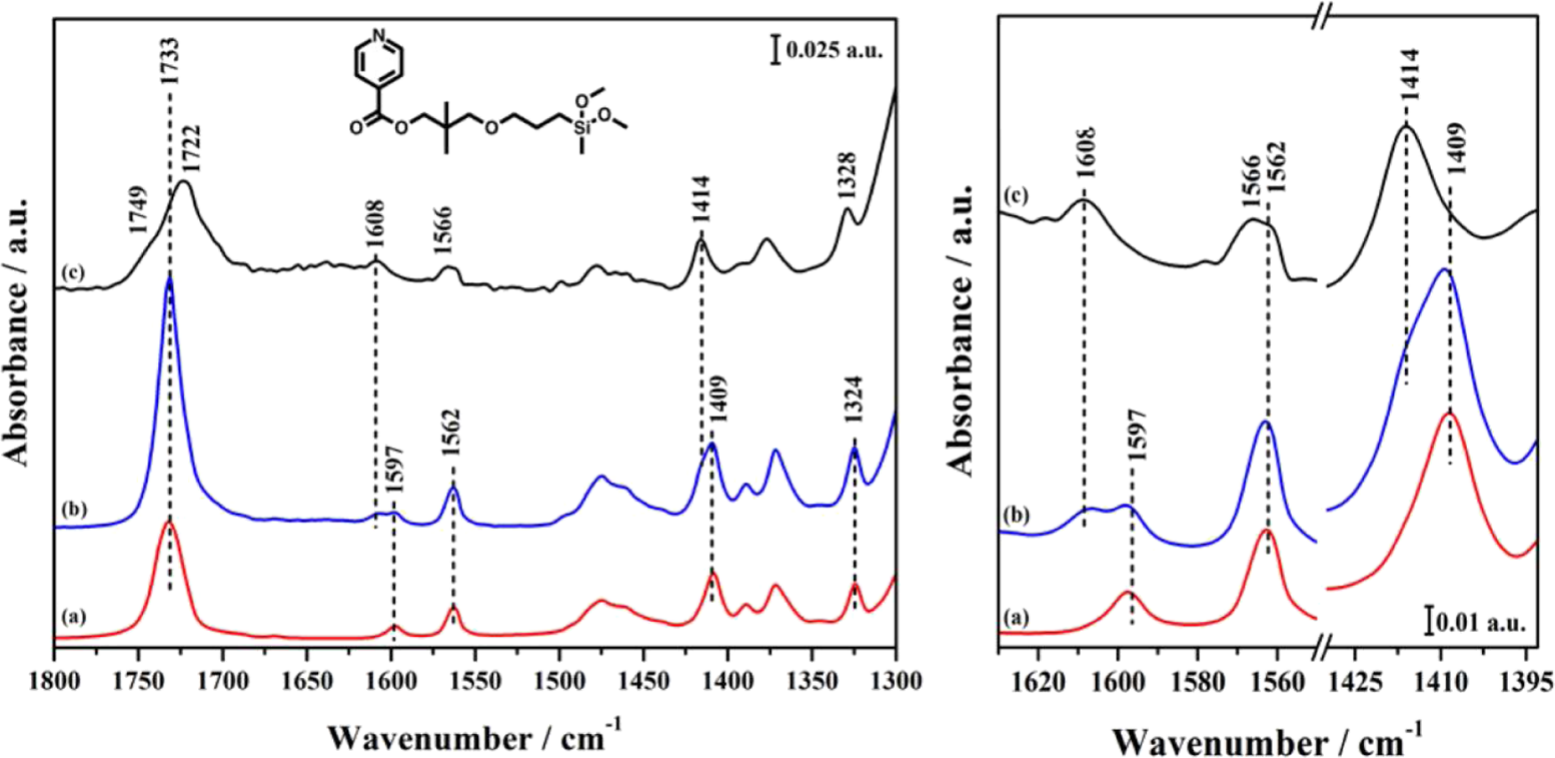
FT-IR spectra of MP-Py in different environments: adsorbed in KBr powder (a, red); adsorbed on MCM-41 (b, blue); grafted on MCM-41, that is, MP-Py-MCM-41 (c, black). Right panel: magnified C–C ring stretching region.

**Figure 6:**
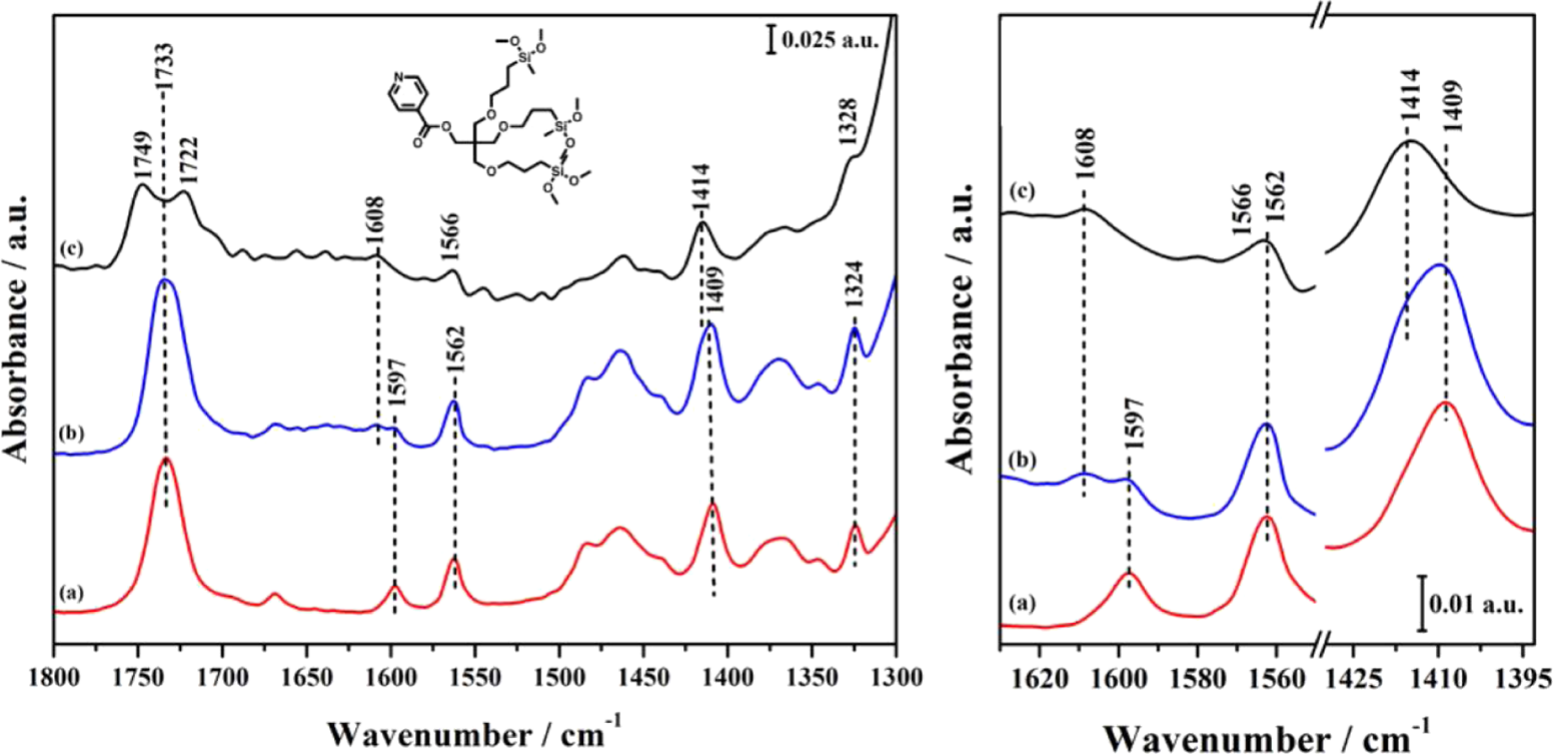
FT-IR spectra of TP-Py in different environments: adsorbed in KBr powder (a, red); adsorbed on MCM-41 (b, blue); grafted on MCM-41, that is, TP-Py-MCM-41 (c, black). Right panel: magnified C–C ring stretching region.

The FT-IR spectra of MP-Py-MCM-41 and TP-Py-MCM-41 are reported in Figure 4; in addition, the spectra of MP-Py and TP-Py simply adsorbed in MCM-41 and KBr are compared to those of hybrids in Figures 5 and 6.

In the high-frequency region (Figure 4A) both hybrid materials show a weak absorption at 3745 cm^–1^ and a broad band between 3700 and 2500 cm^–1^, assigned to the O–H stretching modes of isolated and hydrogen-bonded silanols, respectively. Aromatic and aliphatic C–H stretching modes are detected in 3100–3000 and 3000–2800 cm^–1^ ranges, respectively, the former with a lower intensity, as expected.

The signal due to the C=O stretching mode, associated with the absorptions in the 1722–1750 cm^–1^ range, is particularly interesting for our purposes. As shown in the spectra in Figures 5 and 6, when either MP-Py or TP-Py is adsorbed in the solid matrix, that is, without proceeding to the chemical grafting, this mode produces a single band at 1733 cm^–1^; on the other hand, after the grafting, this band is split into two signals (see Figure 4B), at 1749 and 1722 cm^–1^. In the monopodal hybrid, the band at 1722 cm^–1^ is largely dominant, with just a weak shoulder at 1749 cm^–1^, while in the tripodal system, the two components have the same intensity.

The splitting stems from the interaction with the surface in the hybrid materials: the absorption bands at 1749 and 1722 cm^–1^ can be associated with free and H-bonded carbonyl groups, respectively, the latter indicating a possible interaction with surface silanols. The different aspect of the FT-IR spectra, then, shows that in TP-Py-MCM-41 at least a part of the carbonyl groups remains far from the surface.

The pyridine ring also can interact with silanols if the organic molecules bend toward the surface: the characteristic aromatic C–C stretching modes, in the 1600–1400 cm^–1^ region,^36^ are actually upshifted after the grafting in both MP-Py-MCM-41 and TP-Py-MCM-41. In particular, as seen by comparing the spectra of grafted (Figure 4B) and adsorbed (Figures 5 and 6) pyridine derivatives, the bands at 1409, 1562, and 1597 cm^–1^ move to 1414, 1566, and 1608 cm^–1^, respectively. However, such a shift to higher frequencies is observed for all MP-Py and TP-My modes after the grafting, likely due to the change in the chemical environment rather than to direct interactions with silanols. Then, the shift of pyridine C–C stretching frequencies is not conclusive about the existence or entity of such interactions.

**Figure 7:**
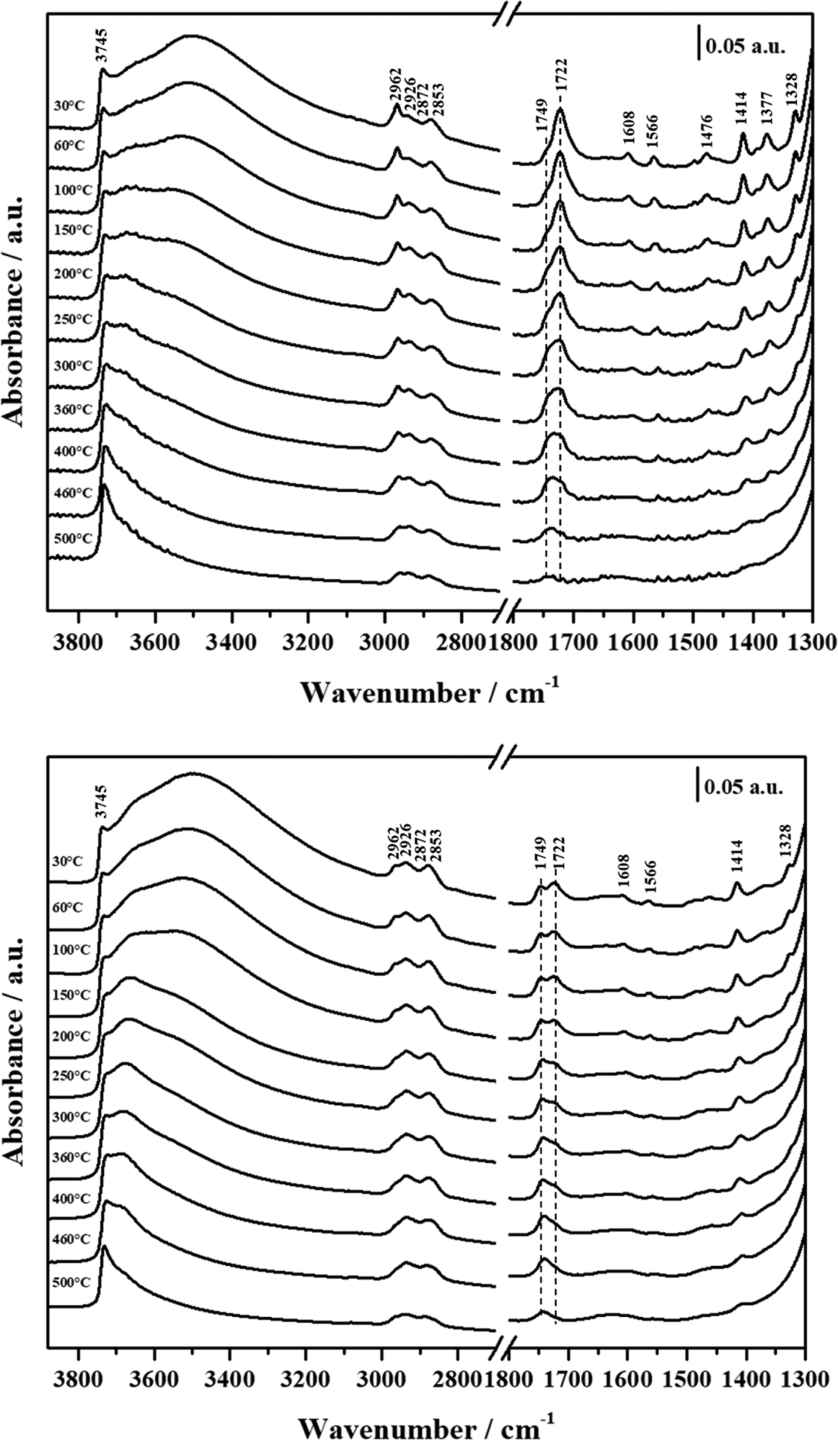
Variable temperature FT-IR spectra of MP-Py-MCM-41 (upper panel) and TP-Py-MCM-41 (lower panel).

Temperature-dependent FT-IR spectra, reported in Figure 7, show a gradual disappearance of the broad band between 3700 and 2500 cm^–1^ when the samples are heated from 30 to 500 °C, due to the loss of physisorbed water and, later, the weakening of H-bonds between silanols. In agreement with TGA, the gradual decomposition of the organic layer starts around 300 °C, as witnessed by the decreasing intensity of aliphatic C–H stretching (3000–2800 cm^–1^), carbonyl stretching (1750–1700 cm^–1^), pyridine ring C–C stretching (1610–1400 cm^–1^). Interestingly, as the temperature increases, the component of C=O stretching at 1722 cm^–1^ gradually lowers and eventually disappears, while the band at 1749 cm^–1^ becomes predominant in both the hybrid materials. This is consistent with the hypothesis that the lower frequency is associated with carbonyl groups engaged in H-bonding with the surface, which becomes weaker as the molecules extend farther from the silica at higher temperatures.

### Solid-State NMR

**Figure 8:**
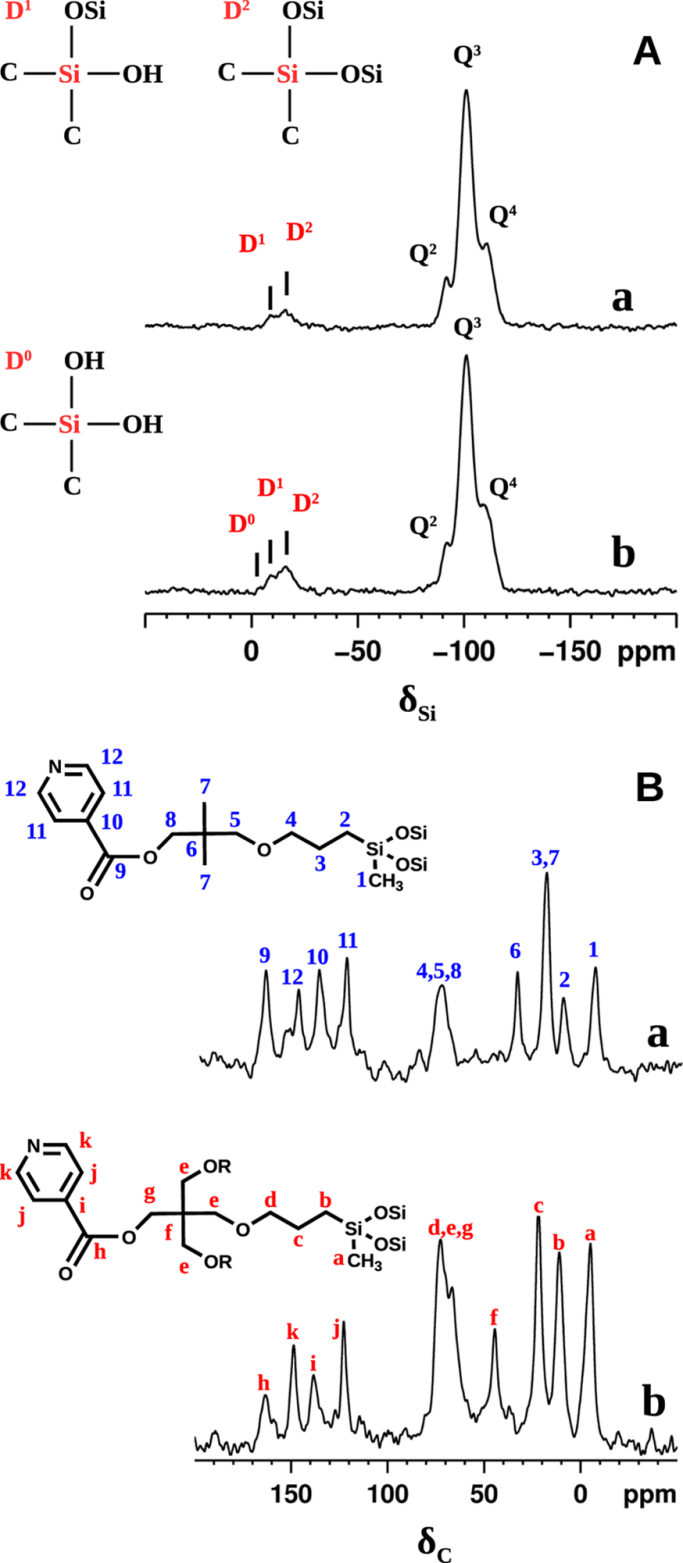
^19^Si (A) and ^13^C (B) CPMAS NMR spectra of MP-Py-MCM-41 (a) and TP-Py-MCM-41 (b) hybrids.

The local environment of silicon atoms in the hybrid materials was investigated with ^29^Si CPMAS NMR, as shown in Figure 8A. The spectra of both MP-Py-MCM-41 and TP-Py-MCM-41 show Q^4^, Q^3^, and Q^2^ resonance peaks (due to tetrahedrally coordinated silicon atoms of the MCM-41 support) at −110, −101, and −91 ppm, respectively, while D^1^ and D^2^ signals (coming from the grafted chain silicon) are found at −9 and −16 ppm, respectively.^37^ In both the spectra, D^2^ is more intense than the D^1^ peak, indicating that didactyl arrangement is preferred (i.e., each organic molecule tends to bind with two siloxane bridges); in the tripodal system, also a low D^0^ signal is detected at −3 ppm, revealing the presence of a small amount of unattached chains, coming either from TP-Py molecules, or from TP-Py-MCM-41 with only one or two siloxane chains grafted to the surface.

The integrity of pyridine derivatives after the grafting was assessed by ^13^C CPMAS NMR (Figure 8B); and the resonance peaks were assigned by comparison with the liquid-state ^13^C spectrum. In both Py-MP-MCM-41 and Py-TP-MCM-41, ^13^C signals can be clearly attributed to the organosiloxane groups, demonstrating the organic chain integrity. A close examination of the spectra reveals that two C nuclei in the hybrids display multiple peaks, associated with different environments, namely carbon in position 12 in MP-Py-MCM-41 (curve a) and carbon in position *h* in TP-Py-MCM-41 (curve b).

**Figure 9:**
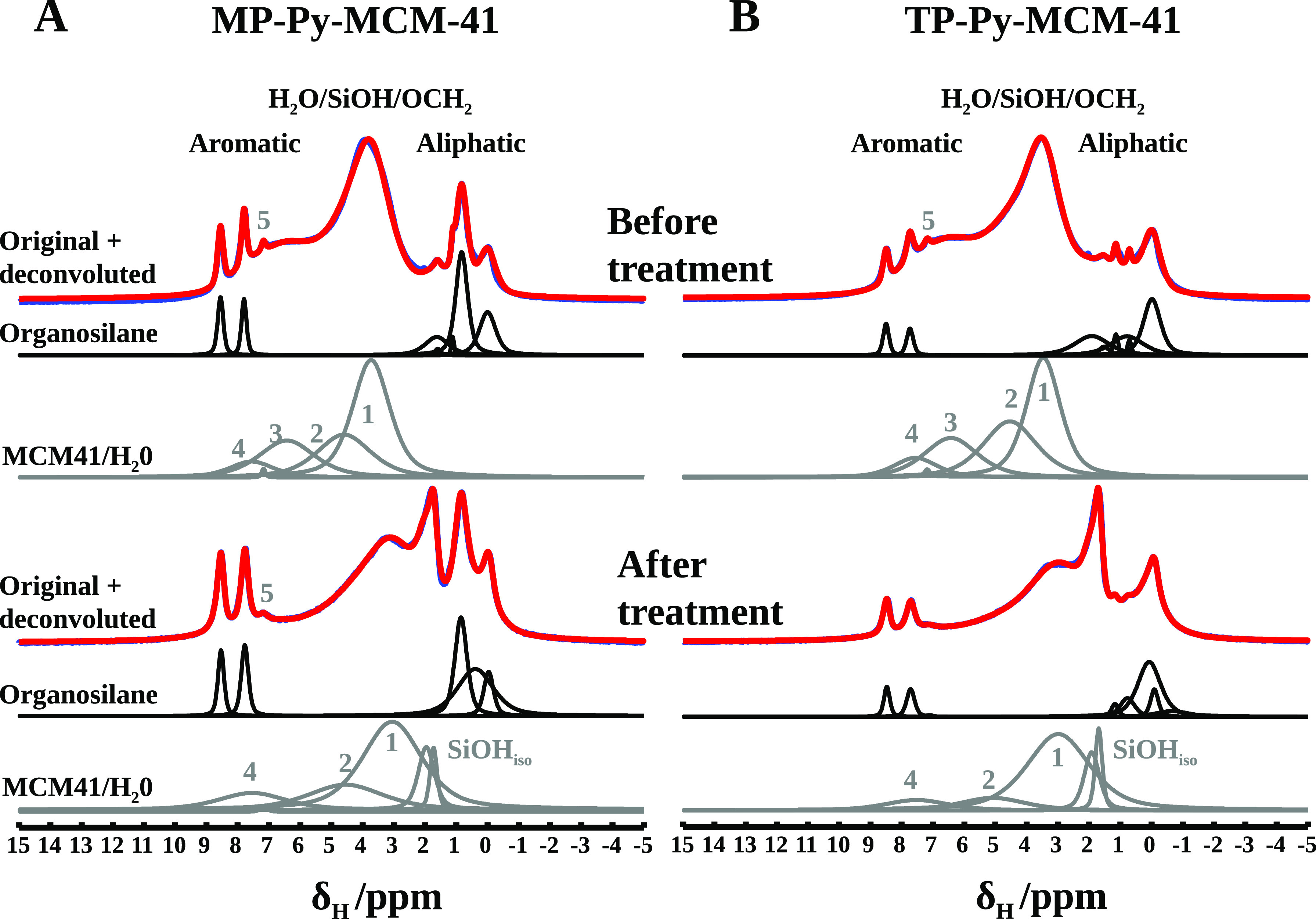
^1^H NMR resonance spectra (red), deconvolution spectra (blue), organic (black), and inorganic (grey) components of MP-Py-MCM-41 (A) and TP-Py-MCM-41 (B) hybrids, as prepared (upper panel) and after mild thermal treatment for degassing (lower panel).

^1^H NMR resonance spectra are reported in Figure 9 for both hybrids, as prepared and after degassing to remove physisorbed water; in the same figure, the spectral deconvolution is presented to separate organosiloxane and silica contributions. In a separate experiment, the ^1^H NMR spectra were recorded after exchange with D_2_O at r.t., in order to remove the signals due to exchangeable protons (i.e., from water and silanols) avoiding the thermal treatment, which could damage the organics as well; besides, it is known that H-bond-accepting groups such as pyridine can form clusters with silanols and water molecules able to resist to mild thermal treatments.^38^

Before the vacuum treatment, the most intense band, around 3.5–5 ppm, is attributed to physisorbed water (marked 1 in the deconvolution curves), H-bonded silanols (marked 2), and also −OCH_2_ from organosiloxane. These components are strongly reduced after degassing, when most of the physisorbed water is removed: at the same time, a sharp peak due to isolated silanols appears around 1.8 ppm.

In the spectra of the D_2_O-exchanged samples, reported in the Supporting Information, the broad subresonance band at 6–8 ppm disappears, confirming that it is related to water and silanol groups: such downfield shifts in the δ values are indicative of strong H-bond interactions, likely due to clusters formed with pyridine nitrogen.

Other contributions from the organic chains are visible at 0 ppm (attributed to a methyl group directly bound to organo-silicon), in the aliphatic region (around 1 ppm), and the sharp peaks at 7.8 and 8.5 ppm, due to the pyridine ring. An addition signal at 7.5 ppm (marked 5) is not straightforwardly assignable, but could be due to silanol or water hydrogens interacting with the carbonyl group, a motif already discussed in the FT-IR study.

In the deconvolution curves before the vacuum treatment, two components marked 3 and 4 are related to water and silanols interacting with the pyridine nitrogen atom: these signals, along with the contribution 5 mentioned above, show that the catalytic center of the organosiloxane interacts with the silica surface, either directly or with the mediation of physisorbed water.

After the thermal treatment for degassing, the component 3 disappears, while 4 is reduced but not eliminated, showing that the former is likely due to water and the latter to silanols, and that the silanol–nitrogen interactions are strong enough to survive after the treatment. Even more interesting for our purpose, the intensity of the band marked 4 reduces more in TP-Py-MCM-41 than in MP-Py-MCM-41 after degassing, a further indication that the tripodal organosiloxane prefers the extended conformation, keeping the pyridine group far from the surface.

### Molecular Dynamics Simulations

Three periodic models of the silica surface were prepared with mono-, di-, and tripodal pyridine derivatives grafted, as described above; all the alkoxysilane chains were anchored to the surface through a didactyl bond, by condensating the organosilicon methoxy groups with silanols placed in suitable positions on the surface.

**Figure 10:**
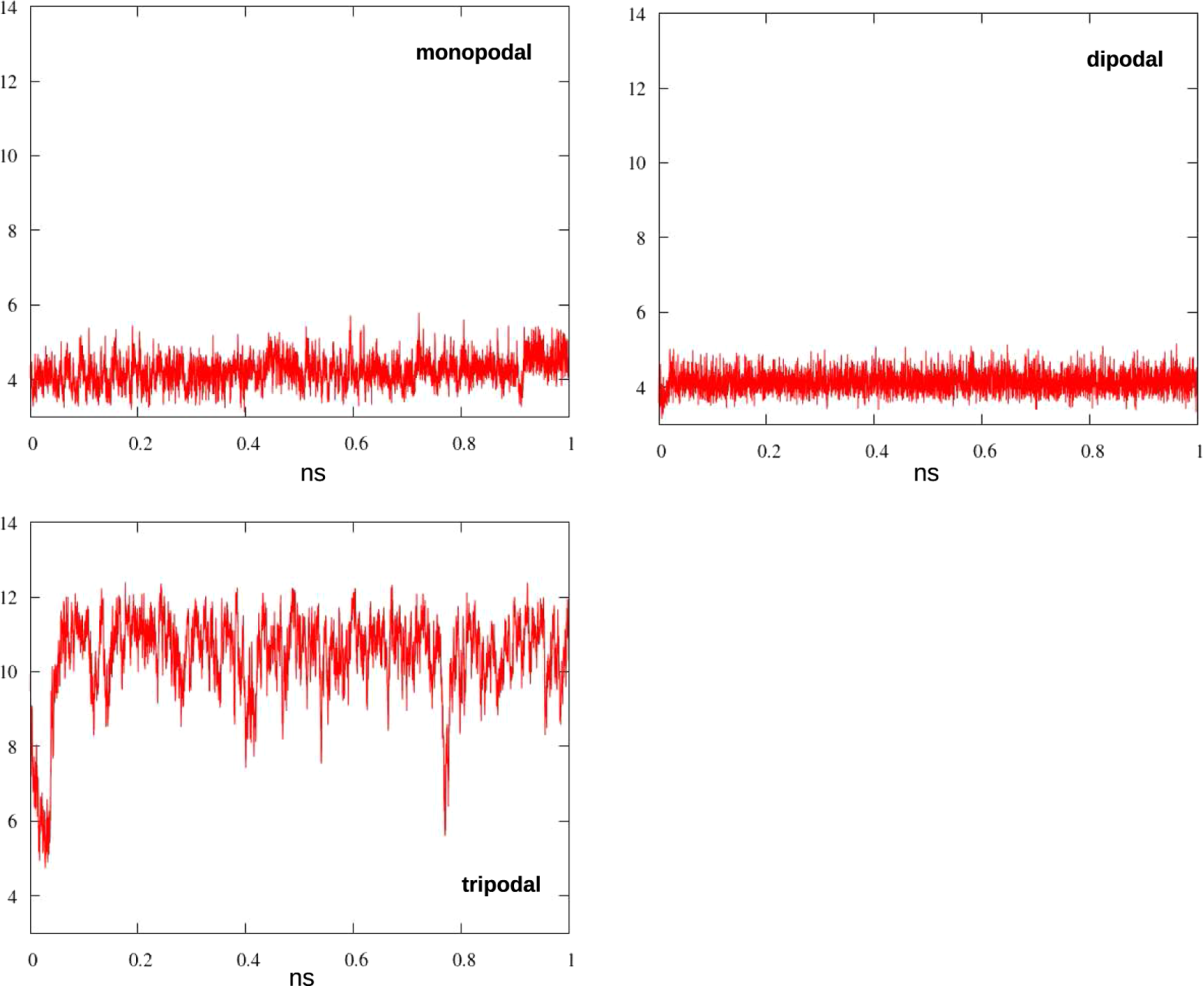
Distance (Å) between pyridine N and the closest Si on the surface along the MD run for mono-, di-, and tripodal hybrid models.

The first set of simulations was performed in vacuum at 298 K, in agreement with the conditions of the physicochemical characterization after degassing, as discussed above: after the equilibration step, the dynamical evolution of the systems was followed for 1 ns, monitoring the distance of the pyridine nitrogen from the closest surface silicon atom. This parameter varied from ca. 4 Å, when the organic chain lies as close as possible to the surface, to ca. 11 Å, when it extends almost perpendicular to the surface: in Figure 10, we show the evolution of the nitrogen-silicon distance during the simulations.

**Figure 11:**
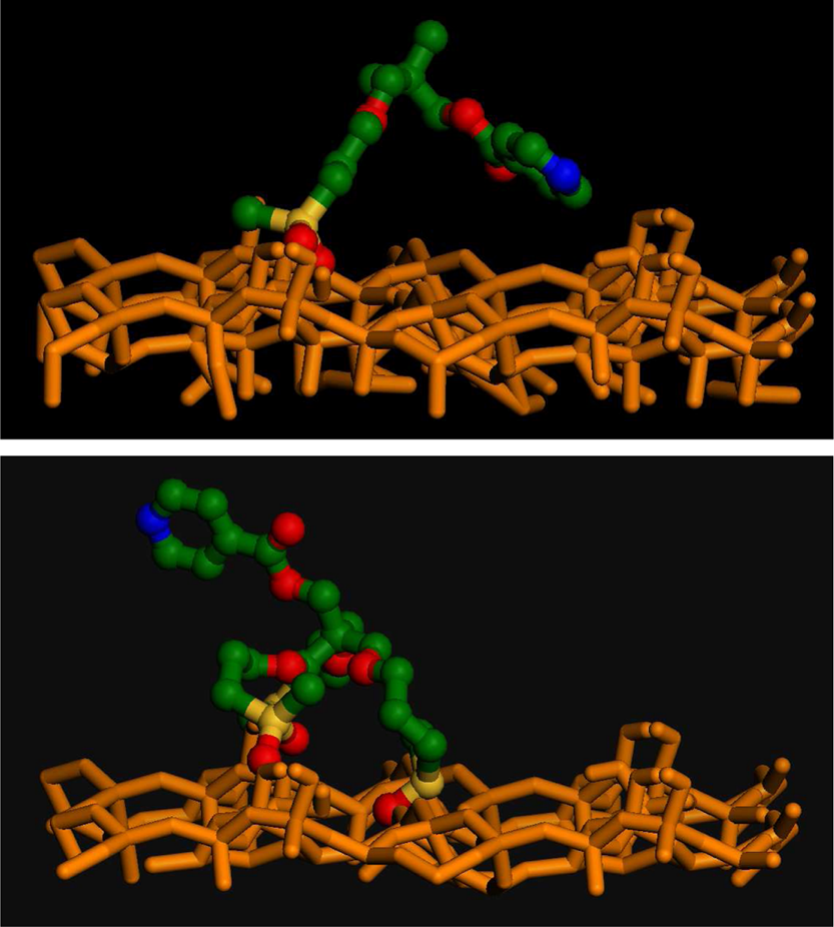
MD frames for MP-Py-MCM-41 (upper) and TP-Py-MCM-41 (lower): lower part of the silica slab and organic hydrogens not shown for clarity; SiO_2_: orange, Si: yellow, C: green, O: red, and N: blue.

The difference among the various grafting schemes is striking: with one or two tethering chains, during the simulation the pyridine spends almost all the time very close to the surface, while in the tripodal system, the catalyst is forced to stay at a much larger distance, always in an extended conformation. To help visualizing the different interface conformations, two representative frames extracted from the MD runs of MP-Py-MCM-41 and TP-Py-MCM-41 are shown in Figure 11. This result is in fair agreement with the FT-IR and NMR findings discussed above, which show the different behavior of monopodal and tripodal hybrids.

**Figure 12:**
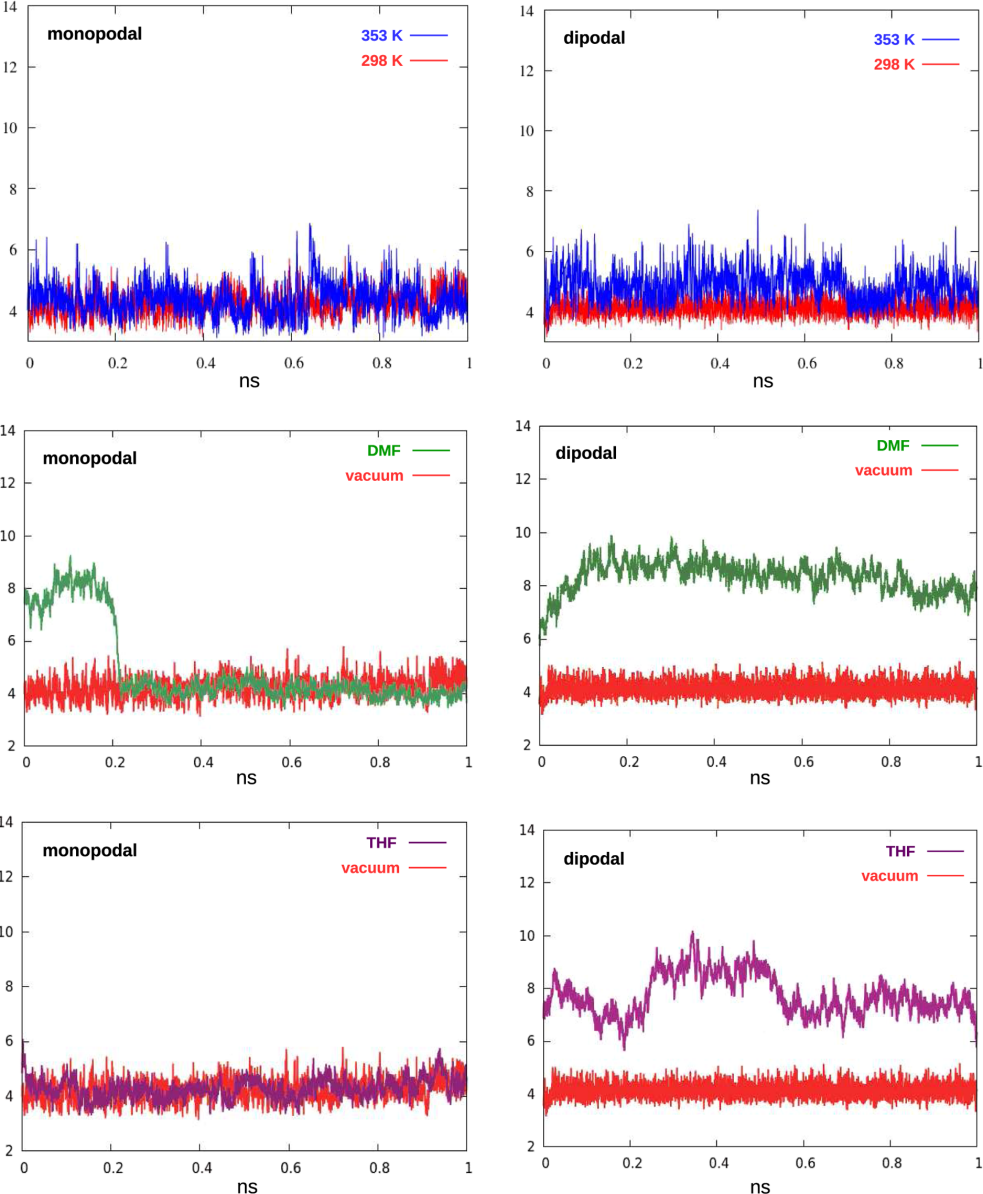
Distance (Å) between pyridine N and the closest Si on the surface along the MD run for monopodal and dipolal hybrids under different conditions: vacuum, 298 K (red); vacuum, 353 K (blue); in DMF, 298 K (green); and in THF, 298 K (purple).

The results in Figure 10 demonstrate that tripodal systems only guarantee that the organic group remains far from the surface at 298 K in vacuum: one can wonder if higher temperatures or the presence of solvents could favor an extended conformation also for the other hybrids. To verify this point, more MD runs were performed on mono- and dipodal systems, either increasing the temperature to 353 K in vacuum, or adding DMF or THF at 298 K, with the results reported in Figure 12.

Even at a substantially higher temperature, thermal motions are not able to surpass the dispersion and H-bond interactions, which pull the organic chain close to the surface, neither in monopodal nor in dipodal systems. On the other hand, the tested solvents act as passivating agents on the surface, competing for the interactions with the organosilica chains: as a result, the dipodal hybrid is removed from its position and forced to extend farther from the surface; the monopodal chain, however, is flexible enough to remain close to the silica even in the presence of a solvent.

We have collected a number of pieces of evidence, to show that the organosilica podality does indeed affect the conformation of the organic/inorganic interface. Only with three grafting points, we are guaranteed that the active center keeps far from the surface, free of interactions, which could hamper its catalytic activity: this is confirmed by the FT-IR and NMR results, as well as by the MD simulations. From the MD results, moreover, we predict that temperature has a little effect in moving monopodal and dipodal systems far from the surface, while the presence of passivating solvents could force dipodal hybrids to leave the surface.

## Conclusions

In this work, we carry out our investigation on the relation between the grafting architecture in organosilica hybrids and the structure of the interface, focusing in particular on the average distance of the catalyst from the inorganic surface.

We had already demonstrated that *silicodactyly* (i.e., the number of siloxane bridges formed by a single organic chain grafted to silica) has a very little effect on the conformation of the organic fragment, which tends to lie down on the surface in all the cases, driven by dispersion and H-bond interactions. Conversely, here we find that *silicopodality* (the number of alkylsiloxane chains used by a single catalytic group to anchor to the surface) can effectively influence the structure of the interface, possibly increasing the catalytic efficiency as the organic part stays farther from the surface, more available to interact with the substrates.

A series of pyridine-substituted derivatives, with one to three alkylsiloxane chains as grafting chains, were used as probes. Mono-, di-, and tripodal systems were synthesized, grafted to MCM-41 mesoporous silica, and characterized: FT-IR and SS-NMR unambigously show that the pyridine heterocyclic ring in the monopodal derivative interacts with the silica surface, while in the tripodal hybrid, such interactions reduce largely, proving that the pyridine is kept farther from the silica.

This finding is confirmed by several MD simulations, involving mono-, di-, and tripodal systems grafted to a silica model: the calculations reveal that only in the tripodal hybrid, the pyridine ring spends almost all the time far from the silica, while in the other systems, it tends to stick on the surface. MD simulations also predict that monopodal and dipodal hybrids lie down on the surface even upon increasing the temperature to 353 K, but adding a solvent (either DMF or THF) that competes for the interactions with the surface, the conformation of the dipodal hybrid changes and the pyridine ring distances from the silica.

These results provide useful information about the relation of grafting and conformation in this kind of interfaces, which should be taken into account when designing hybrid organic/inorganic catalysts.

## Supporting Information Available

Pictures of the silica slab model; XRPD patterns; thermogravimetric curves; ^1^H NMR after D_2_O exchange; details of the organic syntheses; and NMR spectra of new compounds (PDF)
