# Supplementary material for: Predicting the Conformation of Organic Catalysts Grafted on Silica Surfaces with Different Numbers of Tethering Chains: The Silicopodality Concept
Source: J Phys Chem C Nanomater Interfaces. 2021 Sep 17;125(38):21199–210. doi: 10.1021/acs.jpcc.1c06150 (PMC8489525; doi:10.1021/acs.jpcc.1c06150)
Supplement: Supplementary file 1 — jp1c06150_si_001.pdf [file jp1c06150_si_001.pdf]

Electronic Supplementary Material (ESI)

Predicting the Conformation of Organic Catalysts Grafted on Silica Surfaces with Different Numbers of Tethering Chains: the Silicopodality Concept.

Ivana Miletto[1], Chiara Ivaldi[1], Enrica Gianotti[1], Geo Paul[1], Fabio Travagin[2], Giovanni Battista Giovenzana[2,3], Alberto Fraccarollo[1], Davide Marchi[1], Leonardo Marchese[1], and Maurizio Cossi[1]

1) Dipartimento di Scienze e Innovazione Tecnologica (DISIT), Università del Piemonte Orientale, via T. Michel 11, I-15121, Alessandria, Italy

2) Dipartimento di Scienze del Farmaco (DSF), Università del Piemonte Orientale, L.go Donegani 2, I-28100, Novara, Italy

3) CAGE Chemicals srl, Via Bovio 6, I-28100, Novara, Italy

Pag. S2 Picture of the silica slab

Pag. S3 Powder XRD patterns

Pag. S4 TGA and DTG

Pag. S5 ^{1}H-NMR after D_2O exchange

Pag. S6 Details of syntheses and characterization

Pag. S10 NMR spectra of the new compounds

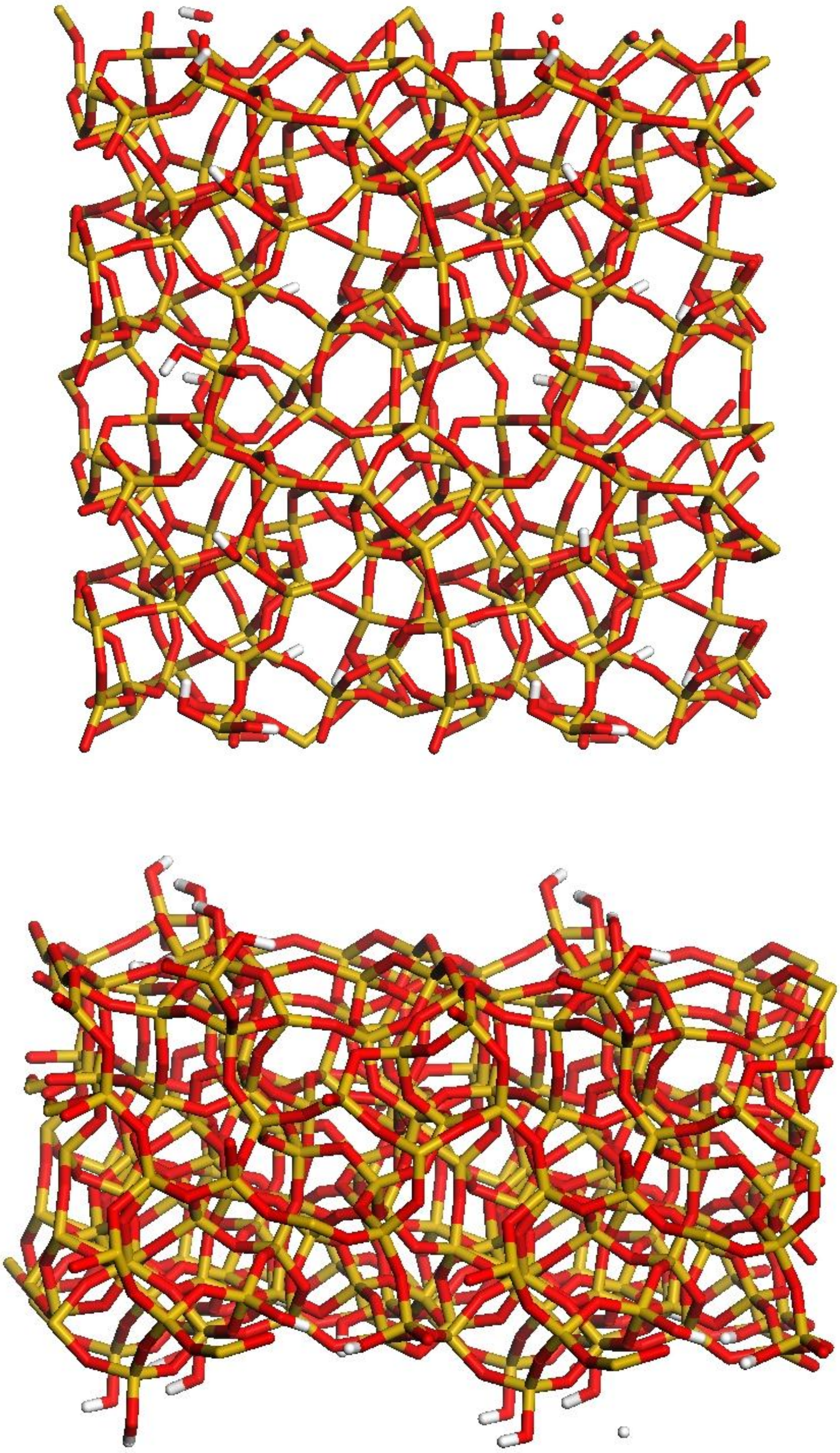

Figure S1. Picture of the silica slab used in the simulations.

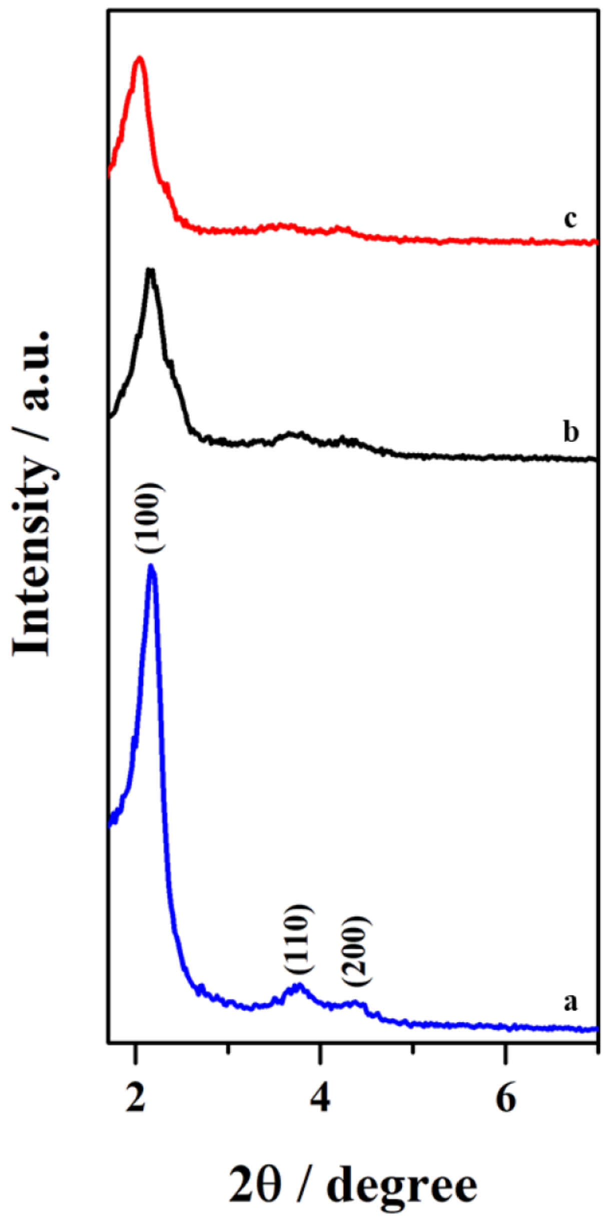

Figure S2. Powder XRD patterns. a) plain MCM-41 (blue curve), b) MP-Py-MCM-41 (black curve), c) TP-Py-MCM-41 (red curve).

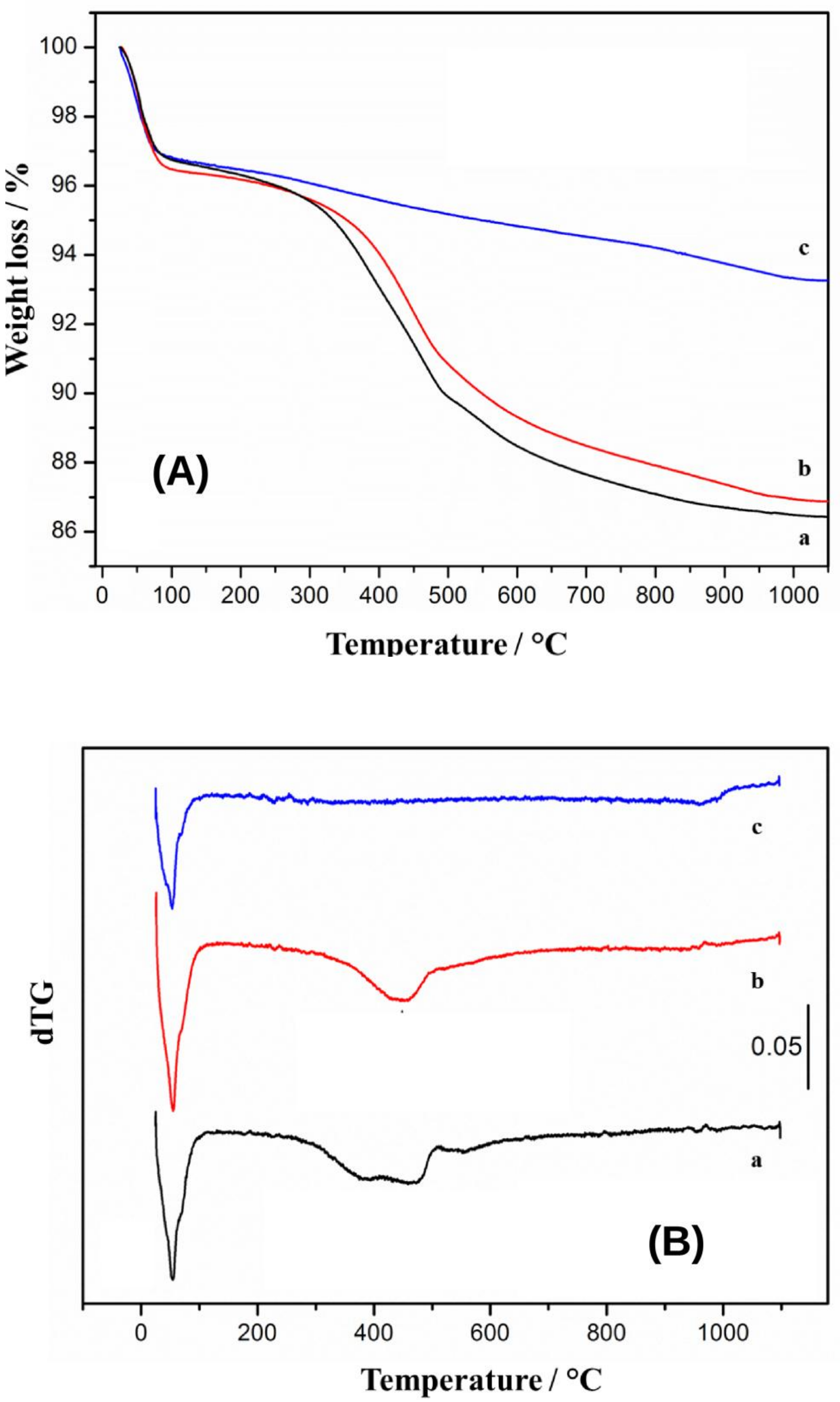

Figure S3. Plot of TGA (A) and DTG (B). MP-Py-MCM-41 (a, black curve), TP-Py-MCM-41 (b, red curve) and plain MCM-41 (c, blue curve).

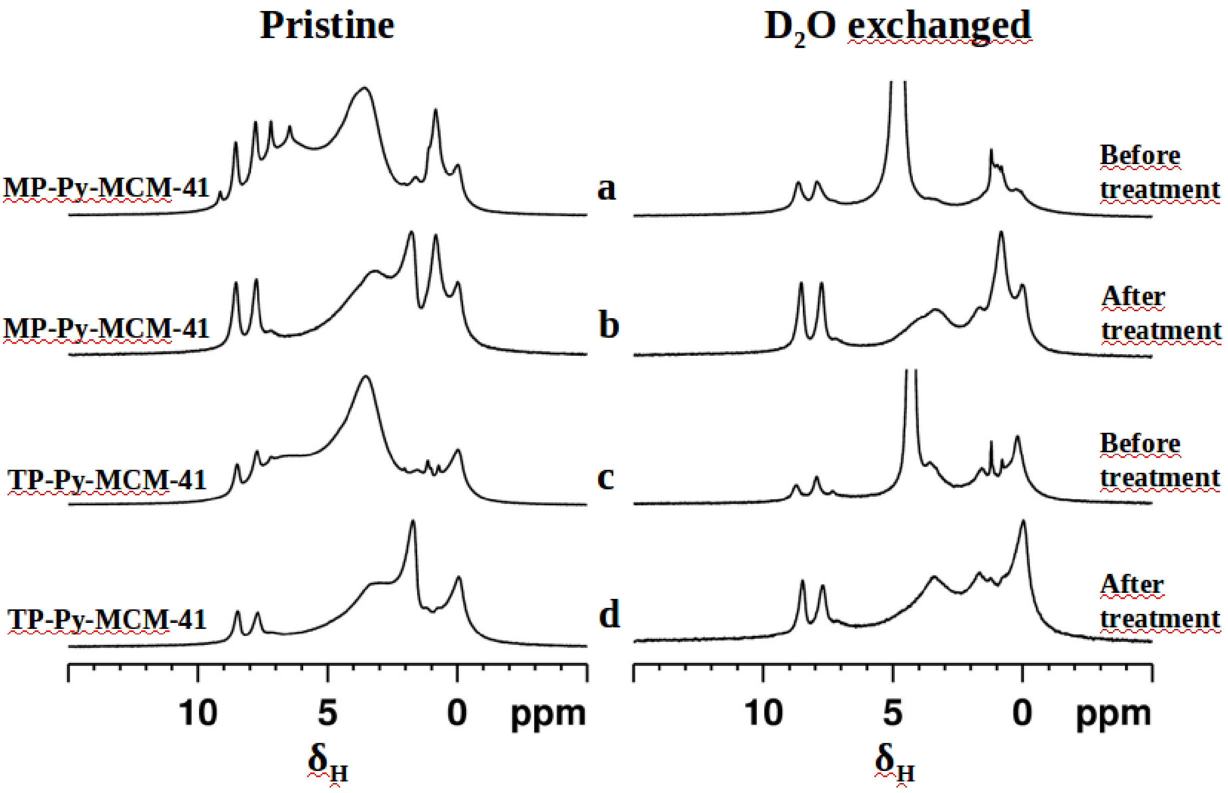

Figure S4. ^{1}H-NMR spectra before and after D_2O exchange.

Details of the organic syntheses and product characterization.

3-(Allyloxy)-2,2-dimethylpropan-1-ol (2)

A dry THF solution (30 mL) of **1** (5.00 g, 48.01 mmol) was added dropwise in 30 min with stirring at room temperature to a suspension of sodium hydride (60% in mineral oil, 2.30 g, 57.61 mmol) in dry THF (10 mL). The resulting mixture was refluxed for 1 h and after cooling to room temperature, allyl bromide (4.98 mL, 57.61 mmol) was added in 1 h to this solution and the mixture was refluxed for 8 h.

The resulting suspension was treated with water and extracted with ethyl acetate. The organic layer was washed with brine, dried over Na_2SO_4 and concentrated *in vacuo* to afford the crude product **2** which was purified by chromatography through a silica column (petroleum ether/ethyl acetate 9:1) as a colourless oil (3.54 g, 51 %).

^{1}H NMR ($CDCl_3$, 300 MHz, 298 K) δ 5.87 – 5.75 (m, 1H), 5.19 (dd, J = 17.2/1.4 Hz, 1H), 5.10 (d, J = 10.4 Hz, 1H), 3.90 (d, J = 5.2 Hz, 2H), 3.36 (d, J = 4.0 Hz, 2H), 3.21 (s, 2H), 2.96 (br s, 1H), 0.85 (s, 6H) ppm.

^{13}C NMR ($CDCl_3$, 75 MHz, 298 K) δ 134.6 (CH), 116.7 (CH_2), 79.0 (CH_2), 72.4 (CH_2), 71.2 (CH_2), 36.2 (C), 21.8 (CH_3) ppm.

MS (ESI$^+$): m/z = 145.12 (100%, $[M+H]^+$). Calc. for $C_8H_{16}O_2$: 144.12.

3-(Allyloxy)-2,2-dimethylpropyl isonicotinate (4)

A dry dichloromethane suspension (25 mL) of **3** (1.85 g, 10.40 mmol) was added dropwise at 0 °C to compound **2** (1.00 g, 6.93 mmol). Then DMAP (2.55 g, 20.79 mmol) was added with stirring and the resulting suspension was stirred at room temperature for 24 h.

The mixture was treated at first with NaOH 0.5 N, the two layers were separated and then the organic phase was washed with HCl 1.0 N. The residual organic layer was washed with brine, dried over Na_2SO_4 and concentrated *in vacuo* to afford the final product **4** as a colourless oil (1.54 g, 89%).

^{1}H NMR (300 MHz, $CDCl_3$, 298 K) δ 8.78 (d, J = 5.0 Hz, 2H), 7.84 (d, J = 5.9 Hz, 2H), 5.91 – 5.79 (m, 1H), 5.24 (dd, J = 17.2/1.7 Hz, 1H), 5.12 (dd, J = 10.5/1.6 Hz, 1H), 4.19 (s, 2H), 3.96 (dt, J = 5.5/1.5 Hz, 2H), 3.27 (s, 2H), 1.03 (s, 6H) ppm.

^{13}C NMR: (75 MHz, $CDCl_3$, 298 K) δ 165.1 (C), 150.6 (CH), 137.9 (C), 135.0 (CH), 123.0 (CH), 116.7 (CH_2), 76.0 (CH_2), 72.4 (CH_2), 71.2 (CH_2), 35.9 (C), 22.2 (CH_3) ppm.

MS (ESI$^+$): m/z = 250.14 (100%, $[M+H]^+$). Calc. for $C_{14}H_{19}NO_3$: 249.14.

3-(3-(Dimethoxy(methyl)silyl)propoxy)-2,2-dimethylpropyl isonicotinate (MP-Py)

Compound **5** (0.85 g, 8.03 mmol) was added to a solution of **4** (0.50 g, 2.01 mmol) in dry diethyl ether (50 mL). Karstedt's catalyst (0.02 g, 0.0524 mmol) was added while stirring at 30°C for 1 h.

The resulting solution was left at room temperature for 34 h, then it was concentrated *in vacuo* to afford the crude **MP-Py** that was purified by chromatography through a silica column (petroleum ether/diethyl ether 8:2 – 6:4) as a colourless oil (0.409 g, 57 %).

^{1}H NMR (300 MHz, $CDCl_3$, 298 K) δ 8.78 (d, J = 5.2 Hz, 2H), 7.84 (d, J = 4.0 Hz, 2H), 4.17 (s, 2H), 3.48 (s, 6H), 3.36 (t, J = 6.6 Hz, 2H), 3.24 (s, 2H), 1.60 (p, J = 7.2 Hz, 2H), 1.02 (s, 6H), 0.60 (t, J = 8.0 Hz, 2H), 0.09 (s, 3H) ppm.

^{13}C NMR (75 MHz, $CDCl_3$, 298 K) δ 165.1 (C), 150.8 (CH), 137.8 (C), 122.9 (CH), 76.7 (CH_2), 74.0 (CH_2), 71.3 (CH_2), 50.2 (CH_3), 36.0 (C), 22.9 (CH_2), 22.2 (CH_3), 9.2 (CH_2), -5.7 (CH_3) ppm.

MS (ESI^+): m/z = 356.19 (100%, $[M+H]^+$). Calc. for $C_{17}H_{29}NO_5Si$: 355.18.

3-(Allyloxy)-2-((allyloxy)methyl)-2-methylpropane-1-ol (7)

A dry THF solution (50 mL) of **6** (5.00 g, 41.61 mmol), was added dropwise in 45 min at room temperature to a suspension of sodium hydride (60% in mineral oil, 4.00 g, 99.88 mmol) in dry THF (90 mL) with stirring. The resulting mixture was refluxed for 1 h and after cooling to room temperature, a dry THF solution (10 mL) of allyl bromide (8.64 mL, 99.88 mmol) was added in 1 h and the mixture was refluxed for 15 h. Then the reaction was quenched with brine, the two layers was separated and the aqueous phase was extracted two times with EtOAc. The organic layers were pooled, dried over Na_2SO_4 and concentrated *in vacuo* to afford the crude product **7**, which was purified by chromatography through a silica column (petroleum ether/ethyl acetate 95:5 – 5:1) as a colourless oil (2.68 g, 32 %).

^{1}H NMR: (300 MHz, $CDCl_3$, 298 K) δ 5.85 – 5.72 (m, 2H), 5.16 (d, J = 17.2 Hz, 2H), 5.06 (d, J = 10.4 Hz, 2H), 3.87 (d, J = 5.5 Hz, 4H), 3.46 (d, J = 1.0 Hz, 2H), 3.35 (dd, J = 9.0/1.0 Hz, 2H), 3.30 (dd, J = 8.9/0.9 Hz, 2H), 3.01 (s, 1H), 0.80 (d, J = 0.9 Hz, 3H) ppm.

^{13}C NMR: (75 MHz, $CDCl_3$, 298 K) δ 134.8 (CH), 116.6 (CH), 74.4 (CH_2), 72.3 (CH_2), 68.6 (CH_2), 40.6 (C), 17.5 (C) ppm.

MS (ESI^+): m/z = 201.15 (100%, $[M+H]^+$). Calc. for $C_{11}H_{20}O_3$: 200.14.

3-(Allyloxy)-2-((allyloxy)methyl)-2-methylpropyl isonicotinate (8)

A dry dichloromethane solution (15 mL) of **3** (2.00 g, 11.23 mmol) was added dropwise to a solution of **7** (1.50 g, 7.49 mmol) in DCM (10 mL). Then a suspension of DMAP (3.66 g, 29.96 mmol) in dry

dichloromethane (25 mL) was added at 0 °C while stirring and then the reaction was stirred at room temperature for 24 h.

The solution was treated with NaOH 0.5 N, the two layers were separated and then the organic phase was washed with HCl 1.00 N and the two layers were separated. The residual organic phase was washed with brine, dried over Na_2SO_4 and concentrated *in vacuo* to afford the product **8** as a colourless oil 2.18 g, 95 %).

^{1}H NMR: (300 MHz, $CDCl_3$, 298 K) δ 8.73 (d, J = 4.4 Hz, 2H), 7.80 (d, J = 4.4 Hz, 2H), 5.88 – 5.74 (m, 2H), 5.20 (d, J = 17.3 Hz, 2H), 5.09 (d, J = 10.3 Hz, 2H), 4.28 (s, 2H), 3.91 (d, J = 3.9 Hz, 4H), 3.36 (s, 4H), 1.03 (s, 3H) ppm.

^{13}C-NMR: (75 MHz, $CDCl_3$, 298 K) δ 164.9 (C), 150.5 (CH), 137.8 (C), 134.8 (CH), 122.9 (CH), 116.6 (CH_2), 72.7 (CH_2), 72.3 (CH_2), 68.3 (CH_2), 40.3 (C), 17.5 (CH_3) ppm.

MS (ESI^+): m/z = 306.17 (100%, $[M+H]^+$). Calc. for $C_{17}H_{23}NO_4$: 305.16.

3-(3-(Dimethoxy(methyl)silyl)propoxy)-2-((3-(dimethoxy(methyl)silyl)propoxy)methyl)-2-methylpropyl isonicotinate (DP-Py)

The Karstedt catalyst (0.0200 g, 0.0524 mmol, 0.02 eq) was dissolved in xilene 1.0 mL in a two necked round bottom flask and then **5** (2.78 g, 26.2 mmol, 10 eq) was added to the mixture and refluxed under nitrogen atmosphere. Then compound **8** (0.8 g, 0.0026 mol, 1 eq) was dissolved in 1.0 mL of xylene and added dropwise to the mixture in 1 h under nitrogen atmosphere. The reaction is refluxed for 6 h and then left at RT for 24 h and checked by TLC (petroleum ether/ethyl acetate 5:5). The mixture is dried under vacuum and purified by column chromatography (petroleum ether/ethyl acetate 7:3) to yield **DP-Py** as a yellow-to-brown oil (0.468 g, 34%).

^{1}H NMR (400 MHz, $CDCl_3$, 298 K) δ 8.77 (d, J = 5.6 Hz, 2H), 7.82 (d, J = 6.1 Hz, 2H), 4.29 (s, 2H), 3.48 (s, 12H), 3.37 – 3.30 (m, 8H), 1.59 (p, J = 7.6 Hz, 4H), 1.03 (s, 3H), 0.59 (t, J = 8.5 Hz, 4H), 0.09 (s, 6H) ppm.

^{13}C NMR (100 MHz, $CDCl_3$, 298 K) δ 165.0 (C), 150.7 (CH), 137.8 (C), 122.9 (CH), 74.0 (CH_2), 73.4 (CH_2), 68.5 (CH_2), 50.3 (CH_3), 40.5 (C), 22.9 (CH_2), 17.6 (CH_3), 9.2 (CH_2), -5.7 (CH_3) ppm.

MS (ESI^+): m/z = 518.15 (100%, $[M+H]^+$). Calc. for $C_{23}H_{43}NO_8Si_2$: 517.25.

3-(Allyloxy)-2,2-bis((allyloxy)methyl)propyl isonicotinate (10)

A dry THF solution (25 mL) of **3** (1.04 g, 5.85 mmol) was added dropwise to **9** (1.00 g, 3.90 mmol) dissolved in 5 mL of dry THF. Then a suspension of DMAP (1.9 g, 15.6 mmol) in dry THF (25 mL) was added at 0 °C while stirring. The reaction was stirred at room temperature for 24 h and then the

mixture was refluxed for 6 h, concentrated *in vacuo* and the residue was taken up with dichloromethane.

The solution was treated with NaOH 0.5 N and the two layers were separated. Then the organic phase was treated with HCl 1.0 N and the two layers were separated. The residual organic phase was washed with brine, dried over Na_2SO_4 and concentrated *in vacuo* to afford the final product **10** as a pale-yellow oil (0.905 g, 64 %).

^{1}H NMR (300 MHz, $CDCl_3$, 298 K) δ 8.77 (d, J = 4.6 Hz, 2H), 7.82 (d, J = 5.4 Hz, 2H), 5.89 – 5.77 (m, 3H), 5.22 (d, J = 18.1 Hz, 3H), 5.11 (d, J = 10.5 Hz, 3H), 4.44 (s, 2H), 3.94 (d, J = 5.7 Hz, 6H), 3.53 (s, 6H) ppm.

^{13}C NMR (75 MHz, $CDCl_3$, 298 K) δ 164.9 (C), 150.6 (CH), 137.8 (C), 134.9 (CH), 123.0 (CH), 116.7 (CH_2), 72.5 (CH_2), 69.3 (CH_2), 65.6 (CH_2), 44.8 (C) ppm.

MS (ESI^+): m/z = 362.20 (100%, $[M+H]^+$). Calc. for $C_{20}H_{27}NO_5$: 361.19.

3-(3-(Dimethoxy(methyl)silyl)propoxy)-2,2-bis((3 (dimethoxy(methyl)silyl)propoxy)methyl)propyl isonicotinate (TP-Py)

Compound **5** (0.71 g, 6.64 mmol) was added to a solution of **10** (0.40 g, 1.11 mmol) in dry diethyl ether (50 mL). Karstedt's catalyst (0.01 g, 0.0262 mmol) was added while stirring at 30°C for 1 h.

The resulting solution was left at room temperature for 36 h, concentrated *in vacuo* to afford the crude **TP-Py**, which was purified by chromatography through a silica column (petroleum ether/diethyl ether 8:2 – 6:4), as a colourless oil (0.274 g, 36 %).

^{1}H NMR (300 MHz, $CDCl_3$, 298 K) δ 8.76 (d, J = 5.1 Hz, 2H), 7.81 (d, J = 5.0 Hz, 2H), 4.40 (s, 2H), 3.47 (br s, 24H), 3.33 (t, J = 6.7 Hz, 6H), 1.57 (p, J = 7.9 Hz, 6H), 0.57 (t, J = 8.4 Hz, 6H), 0.08 (s, 9H) ppm.

^{13}C NMR (75 MHz, $CDCl_3$, 298 K) δ 164.6 (C), 150.7 (CH), 137.9 (C), 123.0 (CH), 74.0 (CH_2), 69.9 (CH_2), 65.8 (CH_2), 50.3 (CH_3), 44.9 (C), 22.9 (CH_2), 9.2 (CH_2), -5.77 (CH_3) ppm.

MS (ESI^+): m/z = 680.33 (100%, $[M+H]^+$). Calc. for $C_{29}H_{57}NO_{11}Si_3$: 679.32.

NMR spectra of the new compounds

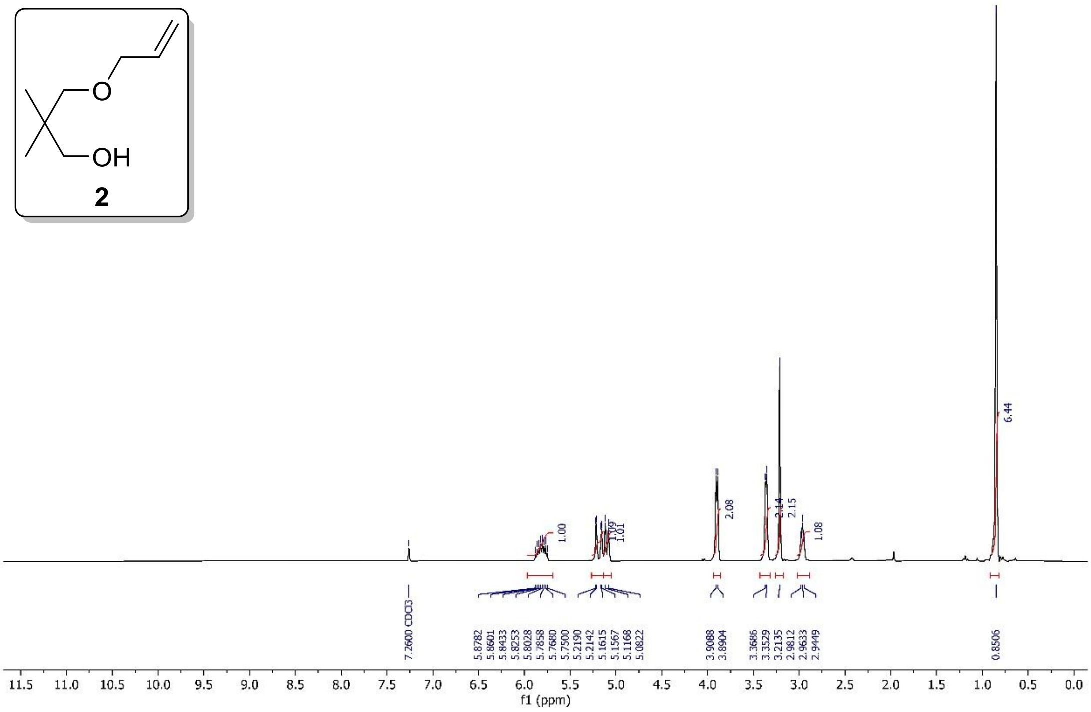

Figure S5 – ^{1}H NMR spectrum of compound **2**

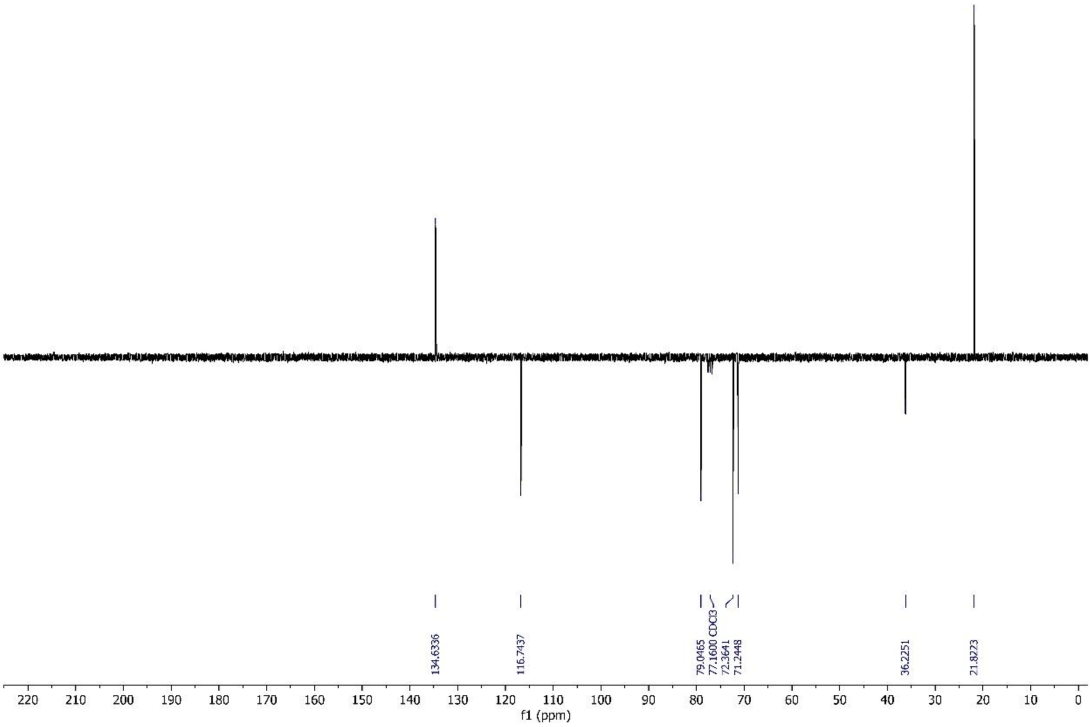

Figure S6 – ^{13}C APT NMR spectrum of compound **2**

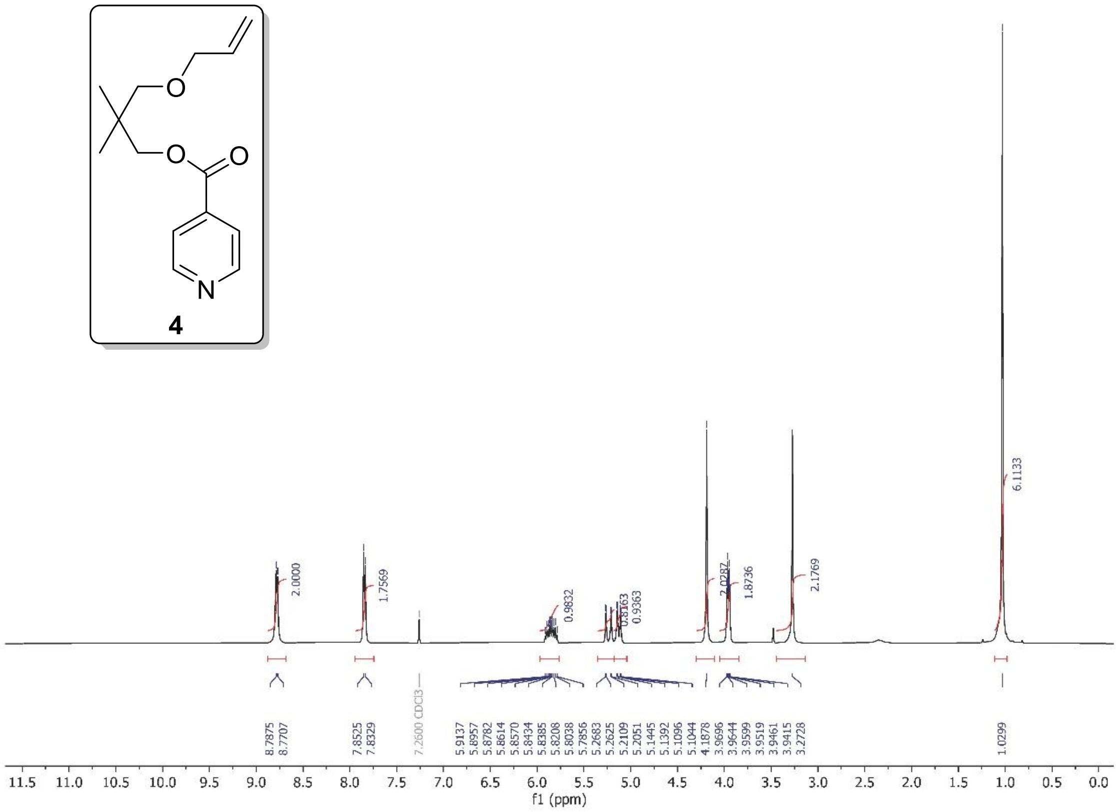

Figure S7 – ^{1}H NMR spectrum of compound **4**

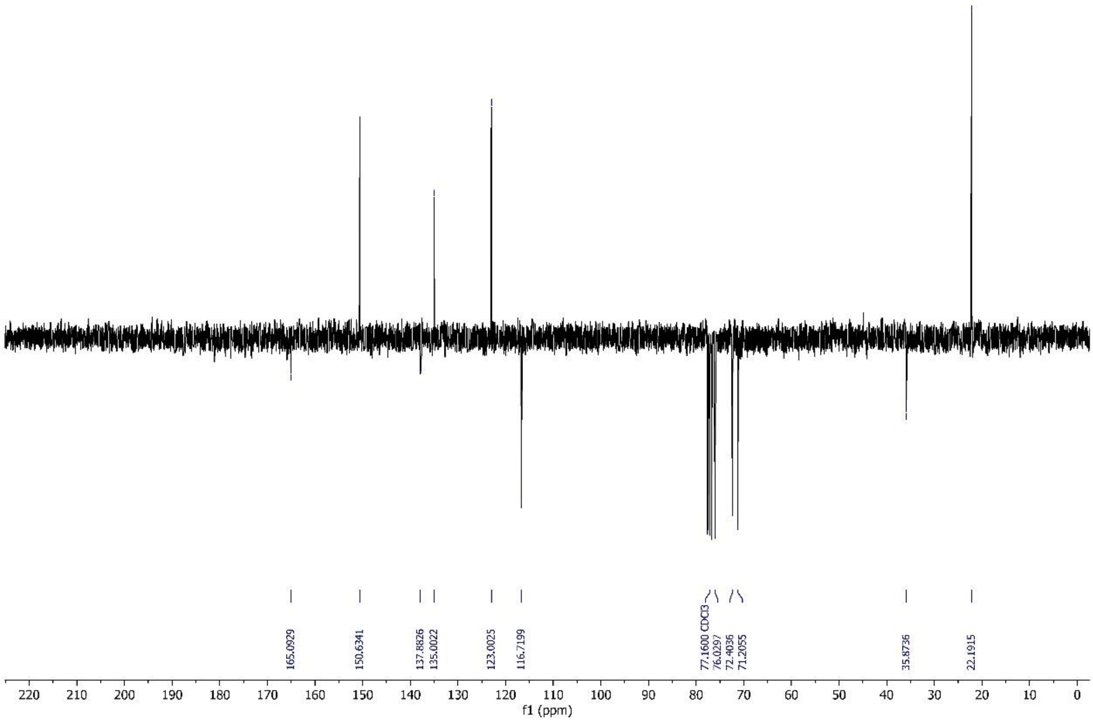

Figure S8 – ^{13}C APT NMR spectrum of compound **4**

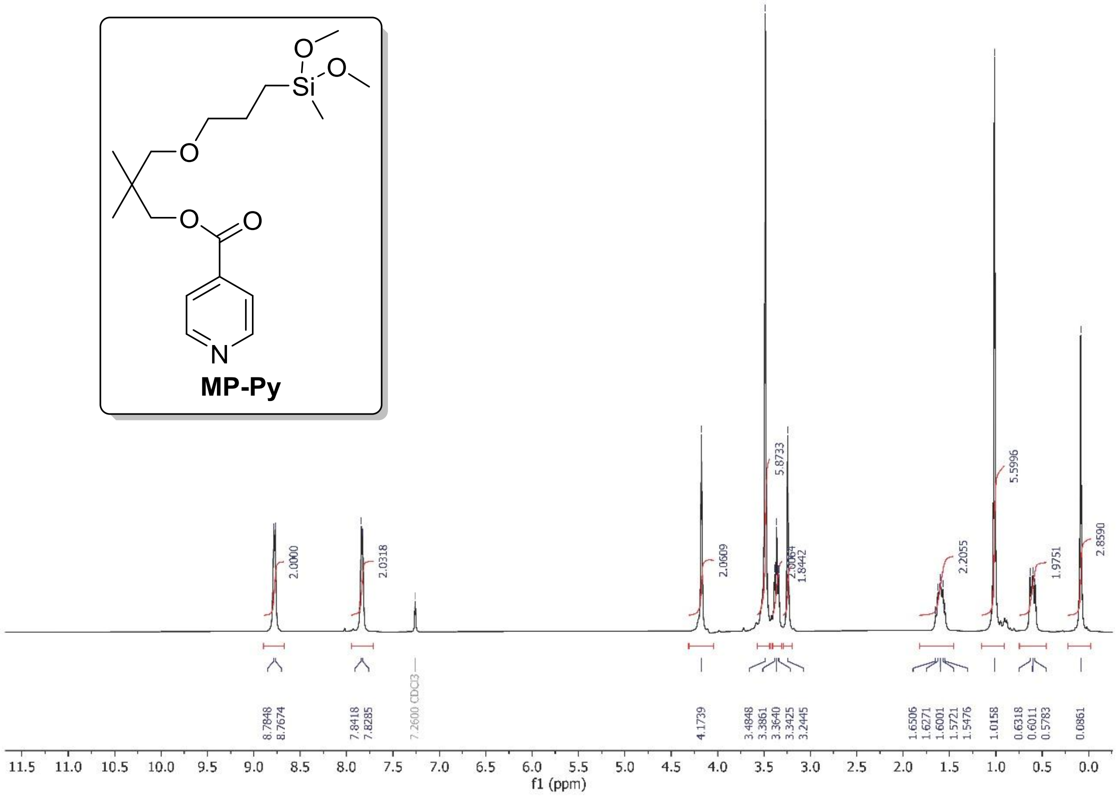

Figure S9 – ^{1}H NMR spectrum of **MP-Py**

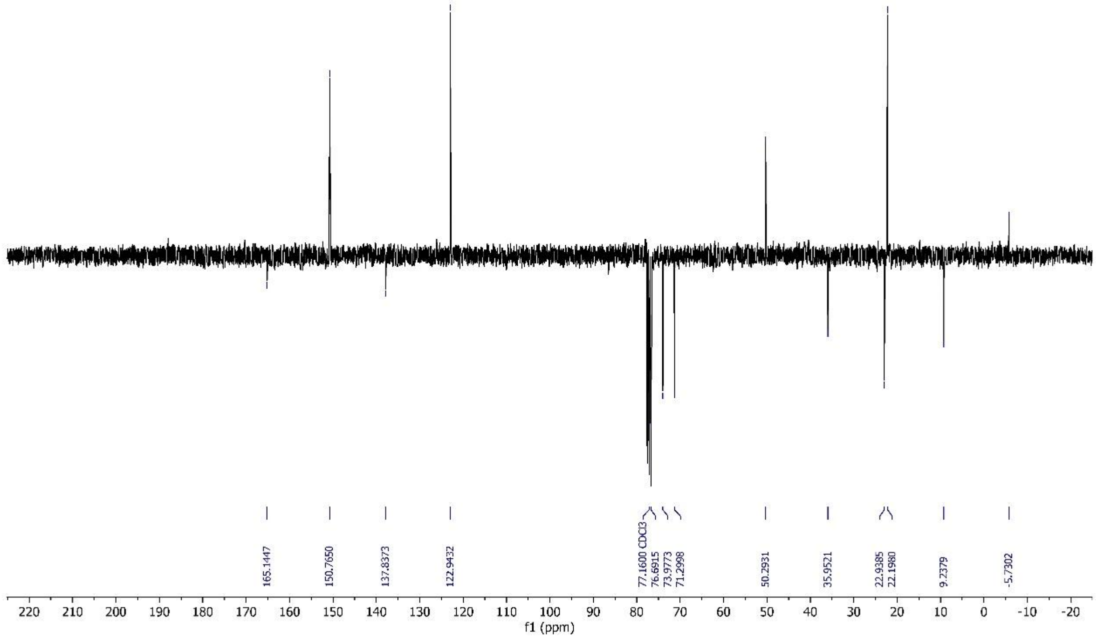

Figure S10 – ^{13}C APT NMR spectrum of **MP-Py**

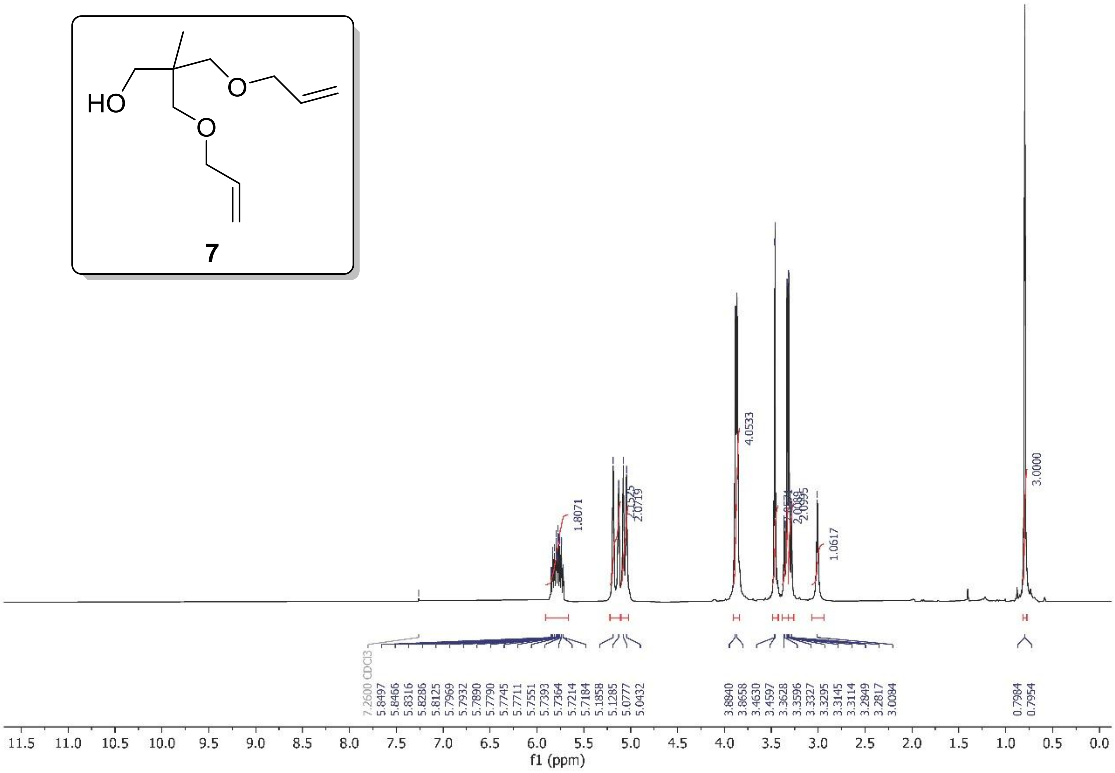

Figure S11 – ^{1}H NMR spectrum of compound **7**

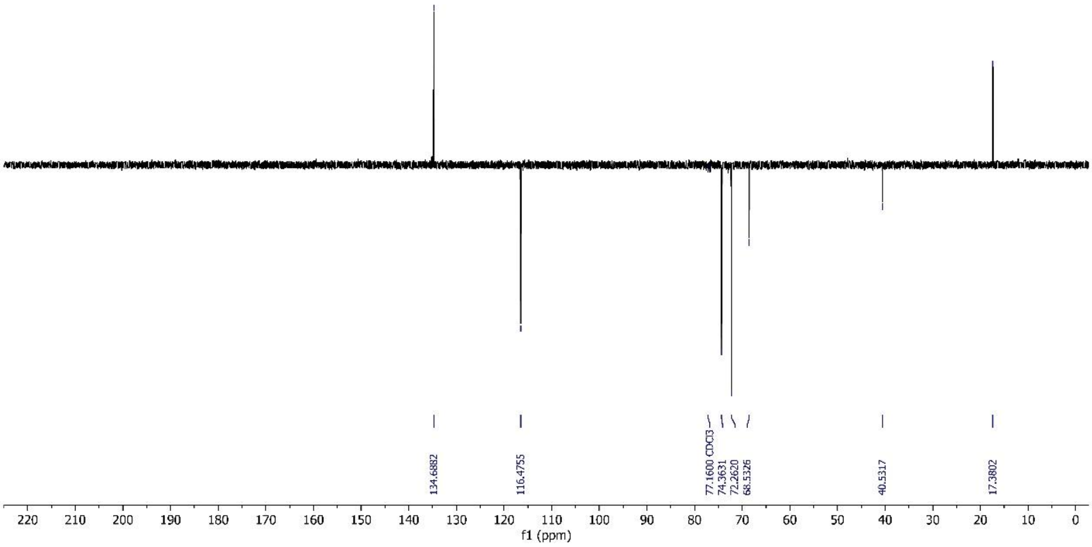

Figure S12 – ^{13}C APT NMR spectrum of compound **7**

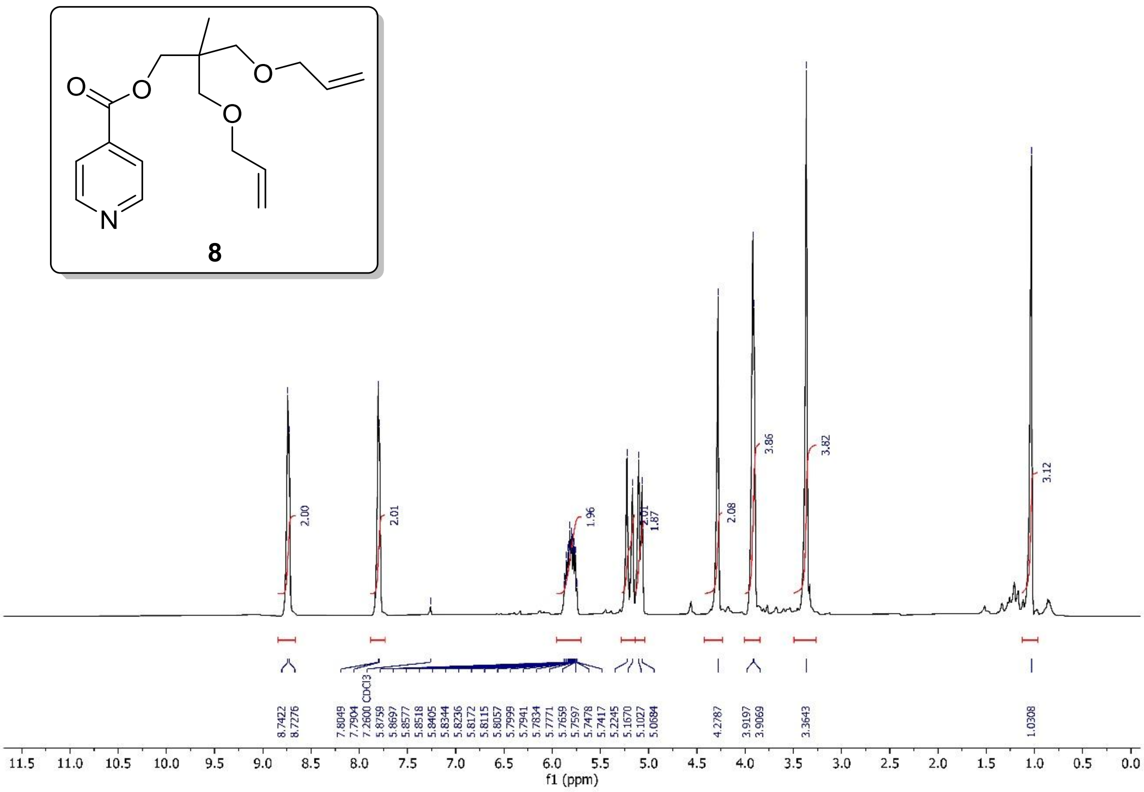

Figure S13 – ^{1}H NMR spectrum of compound **8**

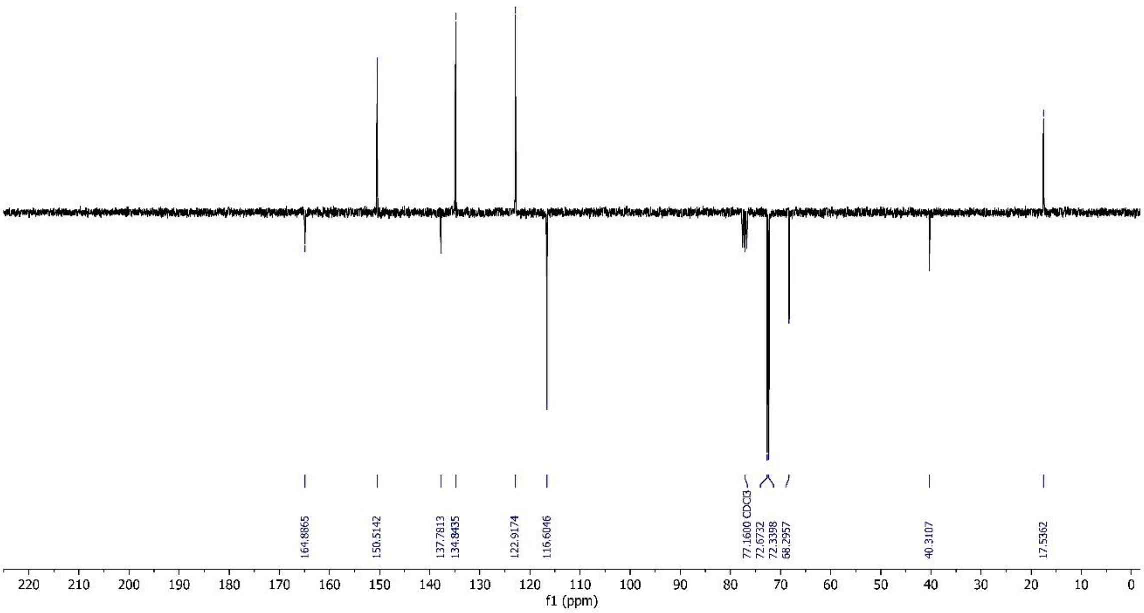

Figure S14 – ^{13}C APT NMR spectrum of compound **8**

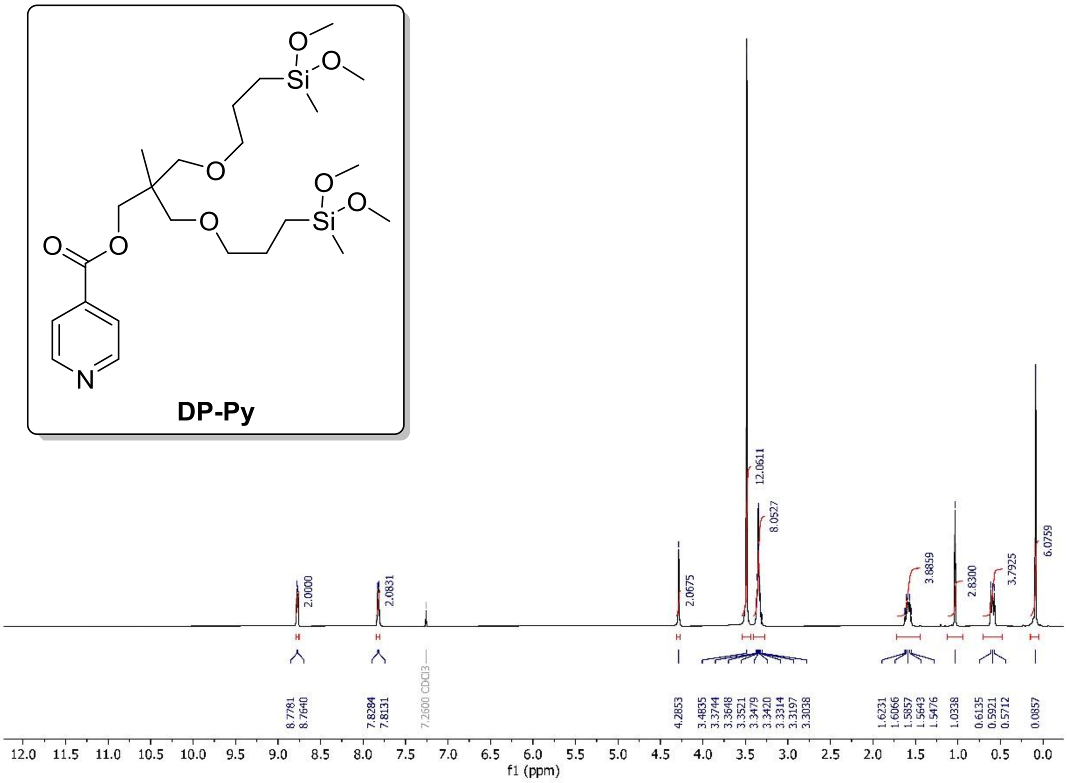

Figure S15 – ^{1}H NMR spectrum of **DP-Py**

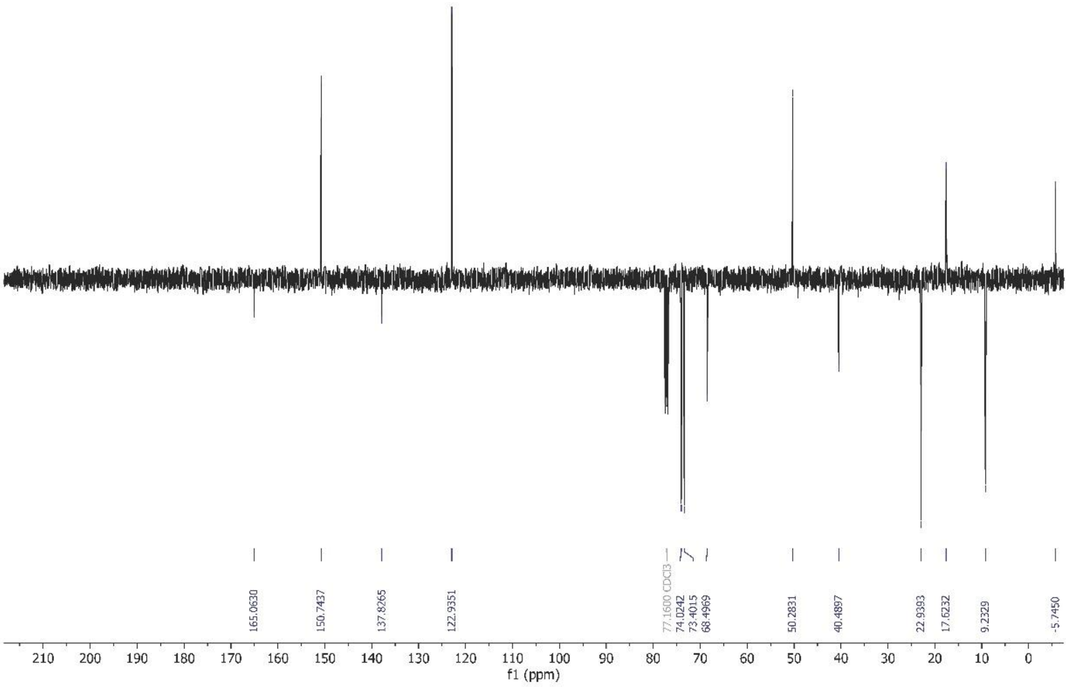

Figure S16 – ^{13}C APT NMR spectrum of **DP-Py**

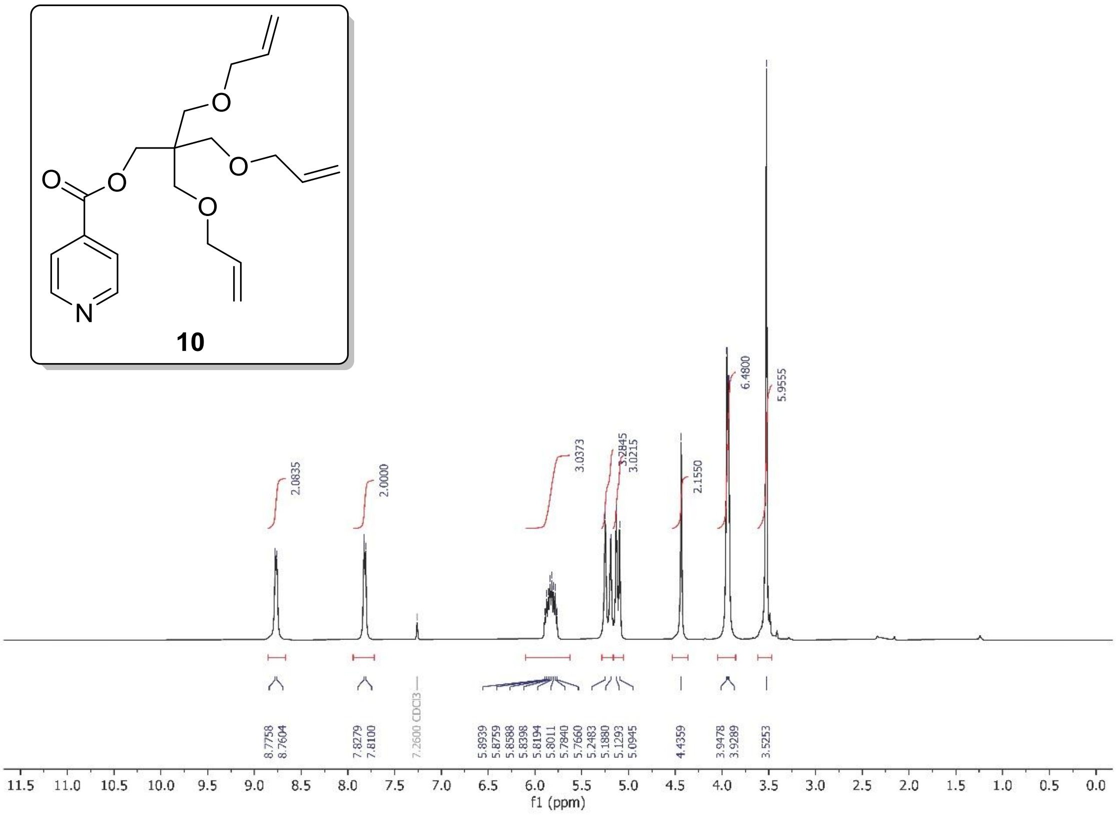

Figure S17 – ^{1}H NMR spectrum of compound **10**

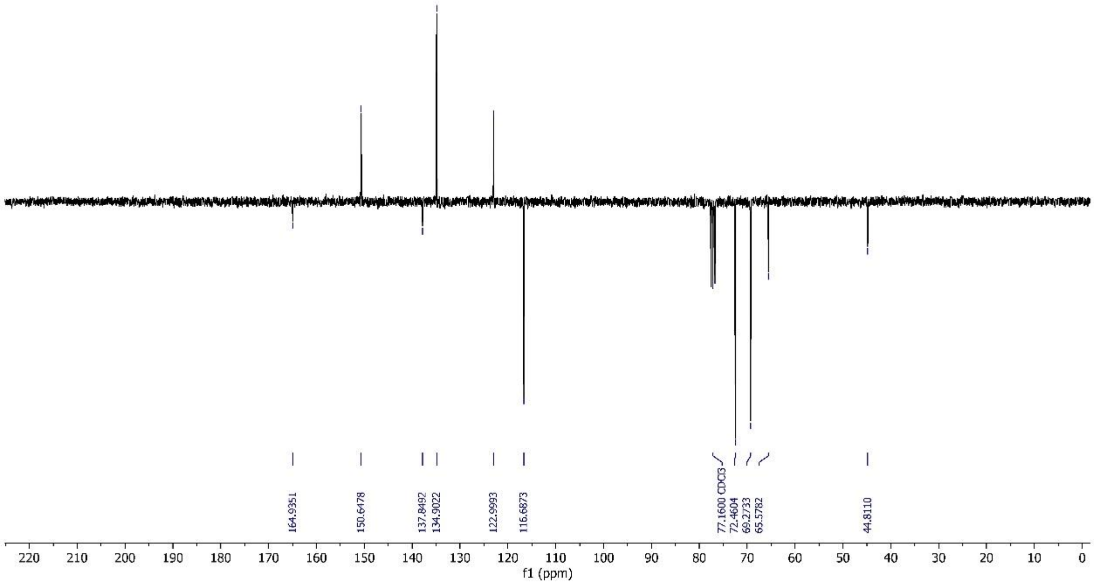

Figure S18 – ^{13}C APT NMR spectrum of compound **10**

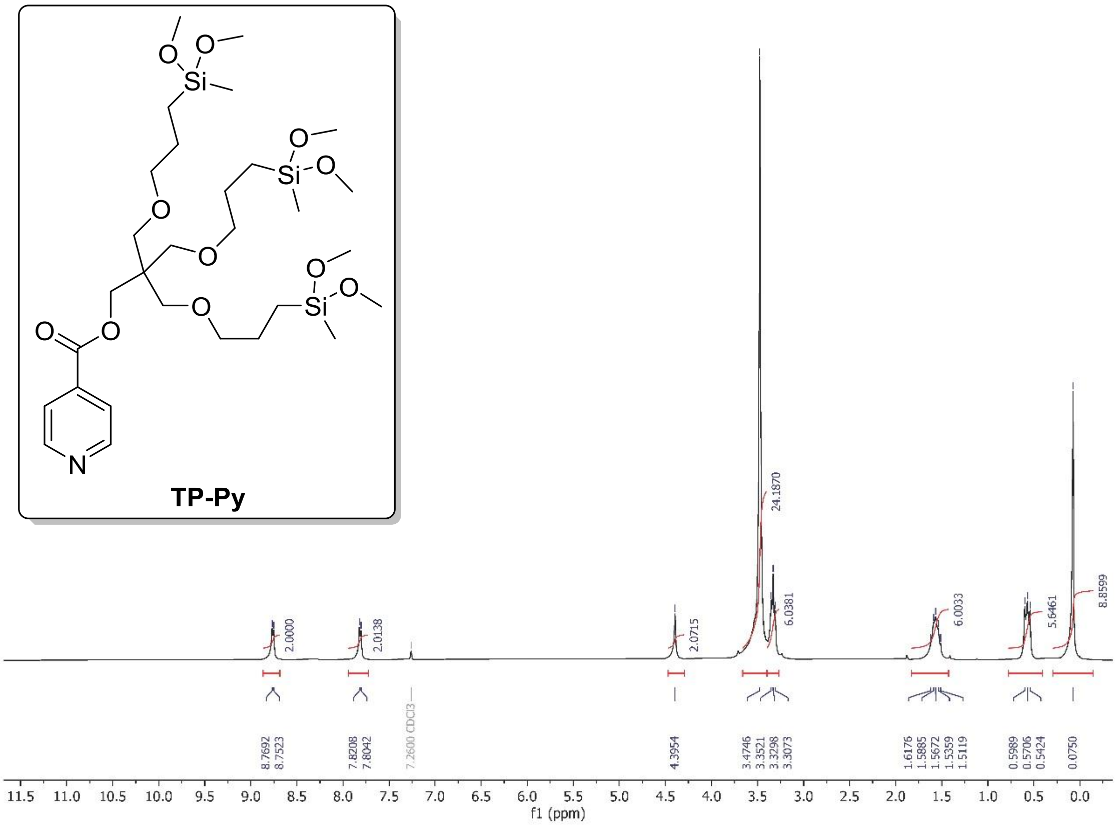

Figure S19 – ^{1}H NMR spectrum of **TP-Py**

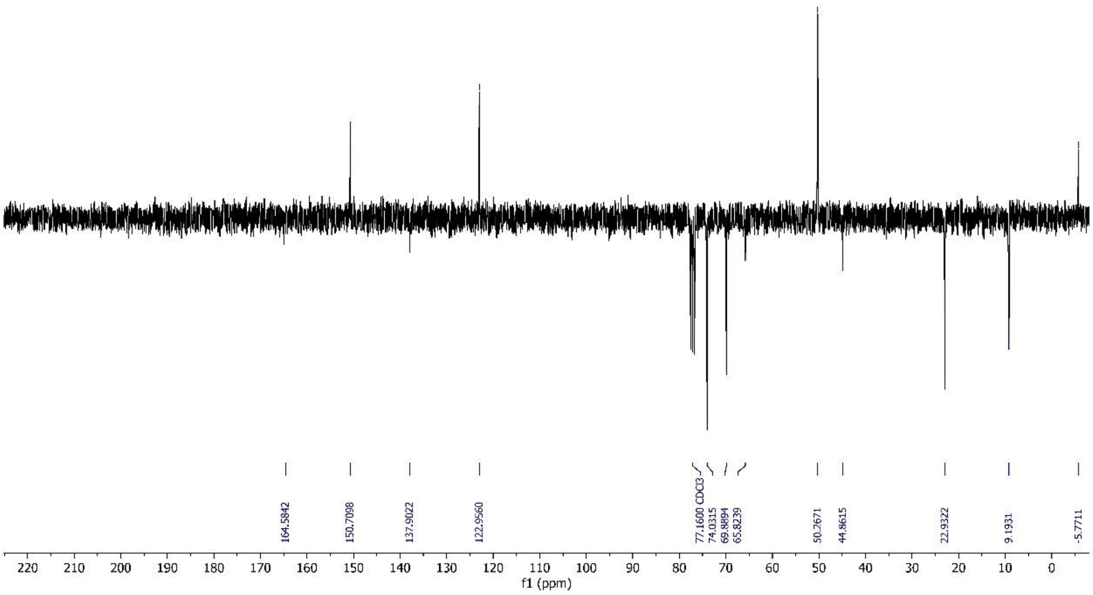

Figure S16 – ^{13}C APT NMR spectrum of **TP-Py**
